# Transporter Protein Expression of Corneal Epithelium in Rabbit and Porcine: Evaluation of Models for Ocular Drug Transport Study

**DOI:** 10.1021/acs.molpharmaceut.3c01210

**Published:** 2024-05-29

**Authors:** Eva Ramsay, Ahmed B. Montaser, Kanako Niitsu, Arto Urtti, Seppo Auriola, Kristiina M. Huttunen, Yasuo Uchida, Heidi Kidron, Tetsuya Terasaki

**Affiliations:** † Drug Research Programme, Division of Pharmaceutical Biosciences, Faculty of Pharmacy, University of Helsinki, 00014 Helsinki, Finland; ‡ School of Pharmacy, University of Eastern Finland, Yliopistonranta 1 C, 70211 Kuopio, Finland; § Department of Molecular Systems Pharmaceutics, Graduate School of Biomedical and Health Sciences, Hiroshima University, 1-2-3 Kasumi, Minami-ku, Hiroshima 734-0037, Japan

**Keywords:** corneal epithelium, transporter protein, targeted absolute quantification, untargeted global proteomics, ocular drug delivery

## Abstract

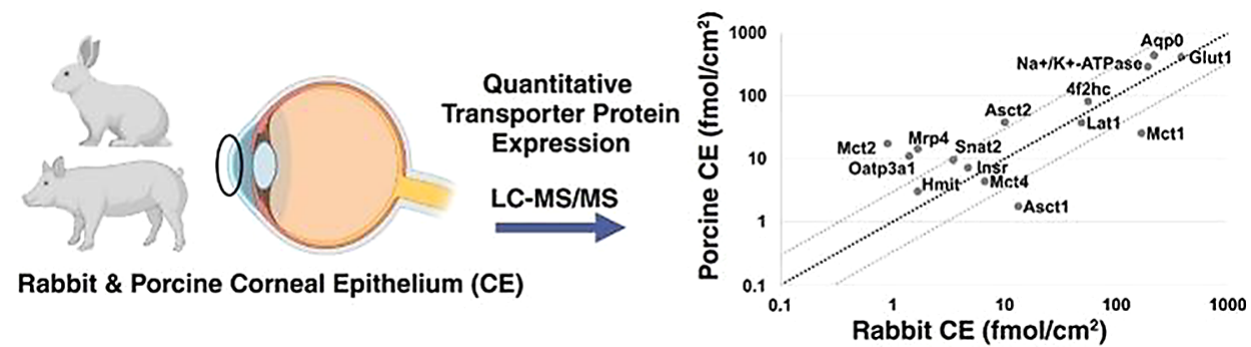

The transcorneal route is the main entry route for drugs to the intraocular parts, after topical administration. The outer surface, the corneal epithelium (CE), forms the rate-limiting barrier for drug permeability. Information about the role and protein expression of drug and amino acid transporter proteins in the CE is sparse and lacking. The aim of our study was to characterize transporter protein expression in rabbit and porcine CE to better understand potential drug and nutrient absorption after topical administration. Proteins, mainly Abc and Slc transporters, were characterized with quantitative targeted absolute proteomics and global untargeted proteomics methods. In the rabbit CE, 24 of 48 proteins were detected in the targeted approach, and 21 of these were quantified. In the porcine CE, 26 of 58 proteins were detected in the targeted approach, and 20 of these were quantified. Among these, 15 proteins were quantified in both animals: 4f2hc (Slc3a2), Aqp0, Asct1 (Slc1a4), Asct2 (Slc1a5), Glut1 (Slc2a1), Hmit (Slc2a13), Insr, Lat1 (Slc7a5), Mct1 (Slc16a1), Mct2 (Slc16a7), Mct4 (Slc16a3), Mrp 4 (Abcc4), Na^+^/K^+^-ATPase, Oatp3a1 (Slco3a1), and Snat2 (Slc38a2). Overall, the global proteomics results supported the targeted proteomics results. Organic anion transporting polypeptide Oatp3a1 was detected and quantified for the first time in both rabbit (1.4 ± 0.4 fmol/cm^2^) and porcine (11.1 ± 5.3 fmol/cm^2^) CE. High expression levels were observed for L-type amino acid transporter, Lat1, which was quantified with newly selected extracellular domain peptides in rabbit (48.9 ± 11.8 fmol/cm^2^) and porcine (37.6 ± 11.5 fmol/cm^2^) CE. The knowledge of transporter protein expression in ocular barriers is a key factor in the successful design of new ocular drugs, pharmacokinetic modeling, understanding ocular diseases, and the translation to human.

## Introduction

Topical drug administration is the most prevalent administration route in ocular drug treatment.^1,2^ Diseases in the anterior part of the eye, such as glaucoma, iritis, inflammation, and dry eyes, are mainly treated with eye drops. In the treatment of iritis and glaucoma, the drug needs to reach the anterior chamber to be active. The transcorneal path is the main intraocular delivery route for topically applied drugs, and the corneal epithelium (CE) acts as the major rate-limiting absorption barrier.^3−5^ The cornea is composed of three main layers: the inner monolayer endothelium, the stroma, and the most outer multilayered epithelium. The tight barrier properties of the CE and short corneal contact of the drug due to precorneal eliminating factors result in low drug bioavailability of less than 5% in the aqueous humor.^6−11^

Small molecular drugs permeate the cornea by both passive diffusion and active transport with the help of transporter proteins.^12^ Passive diffusion is governed by the physicochemical properties of the drug, such as hydrogen bond formation, Log*D*, and polar surface area.^10,13^ On the other hand, active transport is determined by the drug concentration and affinity of the drug to a transporter, as well as transporter protein expression level and localization. Cellular transporter location, on the apical or basolateral side, and type of transporter (influx or efflux) affect the directionality of the transport. Clinically relevant transporters in the intestine, liver, kidney, and blood–brain barrier play a critical role in drug efficacy and toxicity^14^ and may lead to nonlinear pharmacokinetics, drug–drug interactions, and interindividual response variability. However, the role of transporters in ocular pharmacokinetics is still unclear. Transport protein expression in the cornea has been studied in human, rabbit, and porcine cornea by qualitative methods, such as Western blotting and immunohistochemical staining, but the information is sparse.^12,15^ Many studies, especially with rabbit and porcine tissues, utilize nonspecific antibodies, which may cause false-negative or -positive results.

Ocular pharmacokinetic studies are primarily performed in rabbits *in vivo*.^7,9,11,16,17^ Rabbit is a relevant model due to its eye size, enabling easy handling, and predictions of ocular absorption.^13,18−20^ Porcine, on the other hand, is not used as an *in vivo* model in ocular drug delivery research due to its large size. However, porcine eyes are more easily available than rabbit eyes and are therefore a good option for *ex vivo* studies. Rabbit and porcine eyes have been widely used in *ex vivo* studies on drug permeability and metabolism.^10,13,21−26^ The importance of transporter protein activity has been studied in rabbit cornea using *ex vivo* and *in vivo* methods, but they have mainly focused on efflux transporters using narrow sets of clinically relevant drugs, such as erythromycin, cyclosporine, acyclovir, and prostaglandin analogues.^27−31^

Targeted absolute quantification and untargeted global proteomics approaches have been used previously for studying protein expression of transporters, receptors, and enzymes in ocular barriers.^32−37^ Transporter protein expression levels of OATP1B1 or BCRP,^38^ MRP1 or OCTN1^39^ correlated with the transport activity. Quantitative analyses of transporter and enzyme expression have been conducted in human and porcine retinal pigment epithelium (RPE), as well as in a variety of ocular tissues in humans, respectively.^32,34,36,37^ Nevertheless, quantitative and global proteomics data for the CE are still lacking. This kind of information would improve our knowledge of this rate-limiting barrier and increase our understanding of the ocular animal models. The generated information is expected to improve the interpretation of pharmacokinetic data, give clues for improved ocular drug delivery, and provide tools for predictive kinetic models.

The aim of this study was to characterize transporter protein expression in rabbit and porcine CE by quantitative targeted absolute proteomics and untargeted global proteomics methods for the understanding of possible drug and nutrient absorption after topical administration and to explore interesting enzymes, receptors, and tight-junction proteins in the CE to understand protein expression in healthy animals.

## Materials and Methods

All reagents and materials used in this study were commercially of high-purity analytical grade or ultrapure HPLC grade purchased from Thermo Fisher Scientific (Waltham, MA, USA) and VWR International Oy (Helsinki, Finland). Water was purified by using a Milli-Q Gradient system (Millipore, Milford, MA, USA). The isotopically labeled heavy peptides used were selected by the *in silico* selection criteria reported previously by Kamiie et al.^40^ (Supplementary File 1).

### Tissue Isolation and Cell Fractionation of Corneal Epithelial Cell Lysate and Isolation of Plasma Membrane-Rich Fraction

Rabbit and porcine eyes were obtained from a local slaughterhouse and kept on ice during transportation. The CE was isolated by a sterile scalpel and stored at −80 °C. Both cell lysate and plasma membrane-rich fractions were extracted from the CE using a Minute Plasma Membrane Protein Isolation and Cell Fractionation kit (Invent Biotechnologies, MN, USA) according to the manufacturer’s protocol with slight modification. Briefly, 100 mg of tissue dissociation beads together with isolated porcine or rabbit CE (frozen wet tissue weight: 60–70 mg) was added to the filter cartridge. Then, the tissue was ground with a plastic rod for 1 min in buffer A supplemented with a 1:100 protease inhibitor cocktail (Sigma-Aldrich, MO, USA), mixed a few times by pipetting up and down, incubated on ice with the cap open for 10 min, and centrifuged at 16,000*g* for 1 min. The flow through (i.e., cell lysates) was reintroduced two more times to the filter unit and centrifuged to ensure better lysis. An aliquot (50 μL) of cell lysate was stored at −80 °C, while the rest of the cell lysate was further centrifuged at 700*g* for 1 min. The supernatant (i.e., ruptured membrane and cytosolic components) was transferred to a fresh microcentrifuge tube and centrifuged at 16,000*g* for 30–60 min at 4 °C. The pellet (i.e., total membrane fraction) was resuspended in 200 μL of buffer B and centrifuged at 5000*g* for 5 min at 4 °C. This centrifugation step, in the manufacturer's protocol, was modified from 7800 to 5000*g*, resulting in plasma membrane-rich fractions (including organelle membranes). However, rabbit PM samples, analyzed by the targeted proteomics approach, were centrifuged at 7800*g* for 20 min at 4 °C. The supernatant was transferred to a fresh microcentrifuge tube, and 1.6 mL of cold PBS was added. The sample was mixed by inverting the tube a few times and then centrifuged at 16,000*g* for 60 min at 4 °C. The pellet (i.e., plasma membrane-rich fraction) was dissolved in 0.1 M Tris-HCl buffer and stored at −80 °C. Protein amount in the cell lysate and plasma membrane-rich fractions were measured with the BCA protein assay (Thermo Fisher Scientific, MA, USA).

#### Protein Sample Preparation

First, cell lysates and plasma membrane-rich protein samples of both species were analyzed in an untargeted full scan mode to discover the whole sample proteome and to aid in the selection of unique peptides for absolute protein quantification. Second, the absolute quantity of each selected protein was determined by a Quantitative Targeted Absolute Proteomics (QTAP) approach.^40^

#### Untargeted Proteome Analysis

The cell lysate and plasma membrane-rich fractions of both rabbit and porcine CE were reduced, alkylated, and subjected to two-step enzyme digestions as previously described,^41^ with some modification. Briefly, a total of 50 μg of protein was dissolved in 170 μL of solubilizer (7 M Guanidine hydrochloride, 0.5 M Tris-HCL (pH 8.5), 10 mM EDTA-Na (pH 8.0)). The samples were reduced using an equal amount of dithiothreitol (Merk, Germany) for 60 min at RT while mixing at 700 rpm and alkylated using a 2.5-fold iodoacetamide (Merk, Germany) for 60 min at RT in the dark while mixing at 700 rpm. The reduced and alkylated proteins were precipitated by methanol/chloroform/water (4:1:3) and centrifuged at 15,000*g* for 5 min at 4 °C. The pellet was resuspended in 6 M Urea in 0.1 M Tris-HCl (pH 8.5) and mixed at 700 rpm for 10 min at room temperature. The samples were diluted with 0.1 M Tris–HCl to a final concentration of 1.2 M urea and dissolved completely by intermittent sonication (Branson 3510, Danbury, CT, USA) while cooling down on ice for 30 s until the pellet was dissolved. The dissolved proteins were first digested with endoproteinase LysC (1/100, w/w) (Promega, WI, USA) and 0.05% ProteaseMax (Promega, WI, USA) for 3 h at 30 °C, followed by trypsin (Promega, WI, USA) digestion (1/100, w/w) of protein amount, and incubated at 37 °C for 16 h. The enzyme digestion was quenched by 1% trifluoroacetic acid (Thermo Fisher Scientific, MA, USA), and the digested peptides solutions were filtered and desalted by using SDB-tip and GC-tip (GL Sciences, Japan) or SPE Oasis HLB cartridge (Waters Corporation, USA). The solvents were evaporated by a SpeedVac vacuum concentrator (Thermo Fisher Scientific, MA, USA) or dry-freezing (Lyostar). A total amount of 1 or 10 μg peptides were analyzed by high-resolution mass spectrometers (MS) such as Brukers timsTOF Pro or Thermo Orbitrap Q-Exactive Classic, respectively.

#### Quantitative Targeted Absolute Protein Quantification

The absolute protein quantification was determined based on the ratio between native and spiked heavy labeled peptides as previously described.^40^ The cell lysate and plasma membrane proteins of rabbit and porcine CE were processed using filter-aided sample preparation protocol (FASP) as previously described.^42−44^ The protein digestion steps were performed on a Microcon 30 kDa centrifugal filter (Merk Millipore, Germany). The centrifugation times presented here may have varied depending on the sample. Briefly, a total of 50 μg of protein fraction was loaded on the filter units, the buffer was exchanged with 0.1 M dithiotreitol (Merk, Germany) in UA buffer (8 M Urea in 0.1 M Tris/HCl, pH 8.5), mixed on a thermomixer at 500 rpm for 60 min at RT, and centrifuged at 7000*g* for 15 min. The reduced proteins on the filter were washed twice with UA buffer by centrifugation at 14,000*g* for 15 min at RT. The protein samples were then alkylated using 0.05 M iodoacetamide (Merk, Germany) in UA buffer and mixed on the thermomixer first for 1 min at 600 rpm and then for 20 min at 300 rpm in the dark. After this, the filter unit was centrifuged at 14,000*g* for 10 min. The reduced and alkylated proteins on the filter were then washed twice with UA buffer by centrifugation at 14,000*g* for 15 min at RT. A total volume of 48 μL of ammonium bicarbonate (0.05 M ammonium bicarbonate in Milli-Q water) spiked with 50 fmol of isotopically labeled heavy peptide mixtures (Supplementary File 1), 1:100 (w/w) endoproteinase LysC (Promega, WI, USA) of protein amount, and 0.05% ProteaseMax (Promega, WI, USA) were added to the filters and incubated at 30 °C for 3 h. Calibration curves (0, 0.1, 0.25, 0.5, 1, 5, 20, and 50 fmol) in triplicate were prepared for both animal-specific heavy peptide mixtures and treated similarly to the samples. A total of 1:50 (w/w) TPCK-treated trypsin (Promega, WI, USA) of protein was added, and the mixture was incubated at 37 °C for 16 h. The digested peptides were recovered by centrifugation at 14,000*g* for 10 min followed by another two times peptide elution by using 50 μL of 50% acetonitrile in ammonium bicarbonate buffer. The solvent was evaporated by a SpeedVac vacuum concentrator (Thermo Fisher Scientific, MA, USA) at RT, and the samples were resuspended in 2% acetonitrile/5% formic acid solution in MQ water and mixed on thermomixer for 20 min at 300 rpm at room temperature. A total of 20 μL per sample, containing 25 μg of protein and 25 fmol heavy peptide, were injected into the LC-MS/MS.

### Liquid Chromatography–Mass Spectrometry (LC-MS) Data Acquisition and Analysis

#### Untargeted Proteomics

##### Data Acquisition (Orbitrap Q-Exactive)

The tryptic peptides (10 μg) of plasma membrane-rich fractions of rabbit and pig CE were analyzed by ultraperformance liquid chromatography (UPLC) (Vanquish Flex, Thermo Scientific, Bremen, Germany) coupled with a high-resolution mass spectrometer (MS) (Orbitrap Q-Exactive Classic, Thermo Scientific, Bremen, Germany) following the full scan and data-independent acquisition mode (DIA) as previously described.^45^ The injected peptides were first separated by reversed-phase chromatography composed of an Agilent AdvanceBio Peptide Map 2.1 × 250 mm, 2.7 μm column (Agilent Technologies, Santa Clara, CA, United States), eluents water (eluent A), and acetonitrile (eluent B) acidified with 0.1% (v/v) formic acid. The injection volume was 20 μL, and the flow rate was 0.3 mL/min over a direct gradient of 2% B for 80 min followed by a washing step of 80% B for 7 min before equilibrating the gradient back to 2% B for 3 min. An active gradient of 0–80 min was analyzed in the positive polarity by full MS–SIM mode (resolution: 35,000, AGC target: 3 × 10^6^, maximum injection time: 60 ms, and scan range: 385–1015 *m*/*z*) and DIA mode (resolution: 17,500, AGC target: 2 × 10^6^, maximum injection time: 60 ms, loop count: 25, and isolation window: 24 *m*/*z*).

##### Data Acquisition (timsTOF Pro)

The digested tryptic peptides of rabbit and porcine CE plasma membrane samples were loaded into an Evotip (Evosep) following the manufacturer’s instructions. The samples were analyzed using the Evosep One liquid chromatography system coupled to a hybrid trapped ion mobility quadrupole TOF mass spectrometer (Bruker timsTOF Pro, Bruker Daltonics),^46^ via a CaptiveSpray nanoelectrospray ion source (Bruker Daltonics). A total of 1 μg of samples were separated on an 8 cm × 150 μm column with 1.5 μm C18 beads (EV1109, Evosep) with the 60 samples per day methods (21 min gradient time). Mobile phases A and B were 0.1% formic acid in water and 0.1% formic acid in acetonitrile, respectively. The mass spectrometry analysis was performed in the positive-ion mode using the diaPASEF method^47^ with sample-optimized DIA scan parameters. A pooled sample was run following data-dependent acquisition (DDA) in PASEF mode to be able to adjust the diaPASEF parameters optimally for this specific sample type. To perform sample-specific diPASEF parameter adjustment, the default dia-short-gradient acquisition methods were adjusted based on the sample-specific DDA-PASEF run with the software “tims Control” (Bruker Daltonics). The following parameters were modified for diaPASEF analysis: *m*/*z* range 319.5–1319.5; mass steps per cycle steps 20; and mean cycle time 1.17 s. The ion mobility windows were set to best match the ion cloud density from the pooled sample in the DDA-run.

##### Data Processing

The timsTOF and Orbitrap raw data were processed by DIA-NN software (version 1.8) using the library-free DIA analysis mode.^48^ The MS/MS spectra and retention time were predicted by the DIA-NN algorithm using the current release (2022–09–01) Uniprot reference proteome database for rabbits (UP000001811_9986) and porcine (UP000008227_9823). The predicted MS library was used to search the raw data with 1% precursor and protein group (PG) false detection rate (FDR) thresholds and at least one proteotypic peptide with a length of 7–30 amino acids. The resulting MaxLFQ normalized intensities were used for data evaluation.^49^

#### Targeted Proteomics

##### Data Acquisition

A targeted proteomics approach using LC-MS/MS was employed to measure the absolute quantities of 48 and 59 proteins in the plasma membrane-rich and cell lysate fractions of both rabbit and porcine CE, respectively. A total of 51 and 62 proteotypic rabbit and porcine peptides were specifically selected for quantification as previously described^40^ (Supplementary File 1). The digested and desalted peptides (∼10 μg) were analyzed using a UPLC system coupled with a triple quadrupole mass spectrometer with a heated electrospray ionization source in positive mode (UPLC 1290 and MSD 6495, Agilent Technologies, Santa Clara, CA, USA). The peptides were separated using an AdvanceBio Peptide Map column (Agilent Technologies) with a constant flow rate of 0.3 mL/min and a gradient elution of 0.1% formic acid in water (A) and acetonitrile (B). The gradient included 2–7% B for 2 min, 7–30% B for 48 min, 30–45% B for 3 min, and 45–80% B for 2.5 min before re-equilibrating the column back to 2% B for 10 min.^50^ The quantification of these proteotypic peptides was performed using multiplexed multiple reaction monitoring (MRM) mode and analysis mode. The method was developed by selecting the top 3 highest transitions for each peptide (from single-, double-, or triple-charged y or b fragment ions). All the transitions for rabbit or porcine proteins were followed simultaneously by selecting a 2.5 min retention time window for each transition and 0.5 s of cycle time across the peak. The points generated across each peak ranged from 11 to 90 and 14 to 132 for rabbit and porcine peptides, respectively (acquisition methods in Supporting Information File 1).

##### Data Processing

Protein quantification was determined based on the ratio of at least two transitions of light (native) and heavy (spiked-in) peptides, as outlined in Supporting Information File 1. The transitions that exhibited statistically significant differences in ratios (according to the ANOVA test) among the samples were disregarded. Data was acquired using Agilent MassHunter Workstation Acquisition (Agilent Technologies) and processed by using Skyline software (version 20.1). The lower limits of detection (LLOD) and quantifications were calculated based on a linear calibration curve ranging from 0.1 to 50 fmol and determined according to the criteria listed in Supporting Information File 1.

##### Calculation of Protein Levels in Two Different Units (fmol/μg Protein and fmol/cm^2^ Cornea)

The protein was detected when at least two positive transitions were observed in three to five individual samples of rabbit and porcine cell lysate and rich plasma membrane. The quantitative protein expression level was determined by a one-point calibration. The peak area of each light peptide transition was divided by the respective isotopically labeled heavy peptide transition, to extract the light/heavy ratio (L/H ratio). Then, the protein expression levels (fmol/μg protein) were calculated as follows: (*L/H ratio* × 25 *fmol heavy peptide*)/25 μ*g protein*, where 25 fmol and 25 μg are the amounts of heavy peptides and protein injected for multiplexed MRM analysis, respectively.

The absolute quantitative protein expression was calculated based on 2–3 individual L/H ratios within each sample and the average of 3–5 individual cell lysate and plasma membrane-rich samples, including the standard error of mean (SEM) and standard deviation (SD). Statistical analysis of one-way ANOVA was adopted to ensure the similarity of the individual R/H ratios. The criteria of statistical significance were set at a *p*-value of >0.05. Based on the statistical analysis, individual transitions (R/H ratio) could be removed if they varied significantly (*p* < 0.05). Exceptionally, the quantitative expression level of rabbit Octn1 was calculated by subtracting the expression level of the Octn1/Octn2-specific peptide from the Octn2-specific peptide (Supplementary File 1).

The unit of protein level is important when considering transporter protein function in the cornea epithelium. Therefore, the protein level (fmol/μg protein) was converted to protein level per square centimeter cornea (fmol/cm^2^) for both porcine and rabbit according to eqs 1–4. The weight of isolated CE wet tissue (WT) of one eye (mg of WT/eye; eq 1) was experimentally measured. The mg WT was 7.16 ± 1.65 mg (*n*:8) and 11.21 ± 0.85 mg (*n*:5) for rabbit and porcine eyes, respectively. The surface area of the cornea (cm^2^/eye; eq 1) of a middle-size rabbit (3.0–3.9 kg) was 1.59 ± 0.22 cm^2^/eye^51^ and 1.40 ± 0.19 cm^2^/eye of the porcine (≥27 kg).^52^ In the calculation, the corneal surface area is only used, without considering the number of CE layers in the cornea.

The total amount of wet CE tissue in one sample (mg WT/sample) was divided by the amount of wet tissue per cm^2^ (mg WT/cm^2^) of one eye to extract the total surface area of all CE tissue included in one sample (cm^2^/sample; eq 2). The total amount of protein in one sample was determined with the BCA protein assay kit, and the amount was divided by the total corneal surface area in one sample to determine the total amount of protein per cm^2^ (μg protein/cm^2^; eq 3). The protein amount per cm^2^ was multiplied by the quantitative protein expression level (fmol protein/μg protein) to extract the amount of protein expression in square centimeter CE for both rabbit and porcine (fmol protein/cm^2^; eq 4).
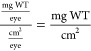
1
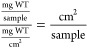
2
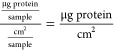
3
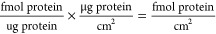
4

## Results

### Plasma Membrane Isolation

Five individual CE cell lysate and plasma membrane-rich fractions were isolated from a pool of 11 rabbit eyeballs (78.1 ± 8.5 mg of wet CE weight) or 7 porcine eyeballs (76.2 ± 27.7 mg of wet CE weight). The protein yields in the plasma membrane-rich fractions were 1.1 ± 0.2 and 0.96 ± 0.33 μg protein/mg wet tissue weight of rabbit and porcine CE, respectively.

The purity of the rabbit and porcine plasma membrane-rich fractions was evaluated using the enrichment factor of membrane markers Na^+^/K^+^ -ATPase and Glut1 in the plasma membrane-rich fractions versus the cell lysate fractions. The enrichment factor by Na^+^/K^+^-ATPase was 21.9- and 29.2-fold and that of Glut1 was 20.7- and 35.6-fold in the rabbit and porcine plasma membrane-rich fractions, respectively. Ljubimov et al. reported that Na^+^/K^+^-ATPase protein is expressed in the basolateral membrane, and Kumagai et al. reported that GLUT1 protein is expressed in the apical and basal membrane of human cornea epithelial cells.^53,54^ As the enrichment factor of Glut1 in the purified plasma membrane-rich fractions was similar to that of Na^+^/K^+^-ATPase, it is suggested that the purified plasma membrane-rich fractions contain a similar ratio of apical and basal membranes to those of whole cell lysate of cornea epithelial cells. The number of detected proteins was also higher in the plasma membrane-rich fractions than in cell lysate fractions, and there were no cases of protein detection in only cell lysate fractions without detection in the plasma membrane-rich fractions (Supplementary File 1). The data support the purity and protein enrichment in the plasma membrane-rich fractions.

### Targeted Proteomics of Rabbit and Porcine CE

**Figure 1:**
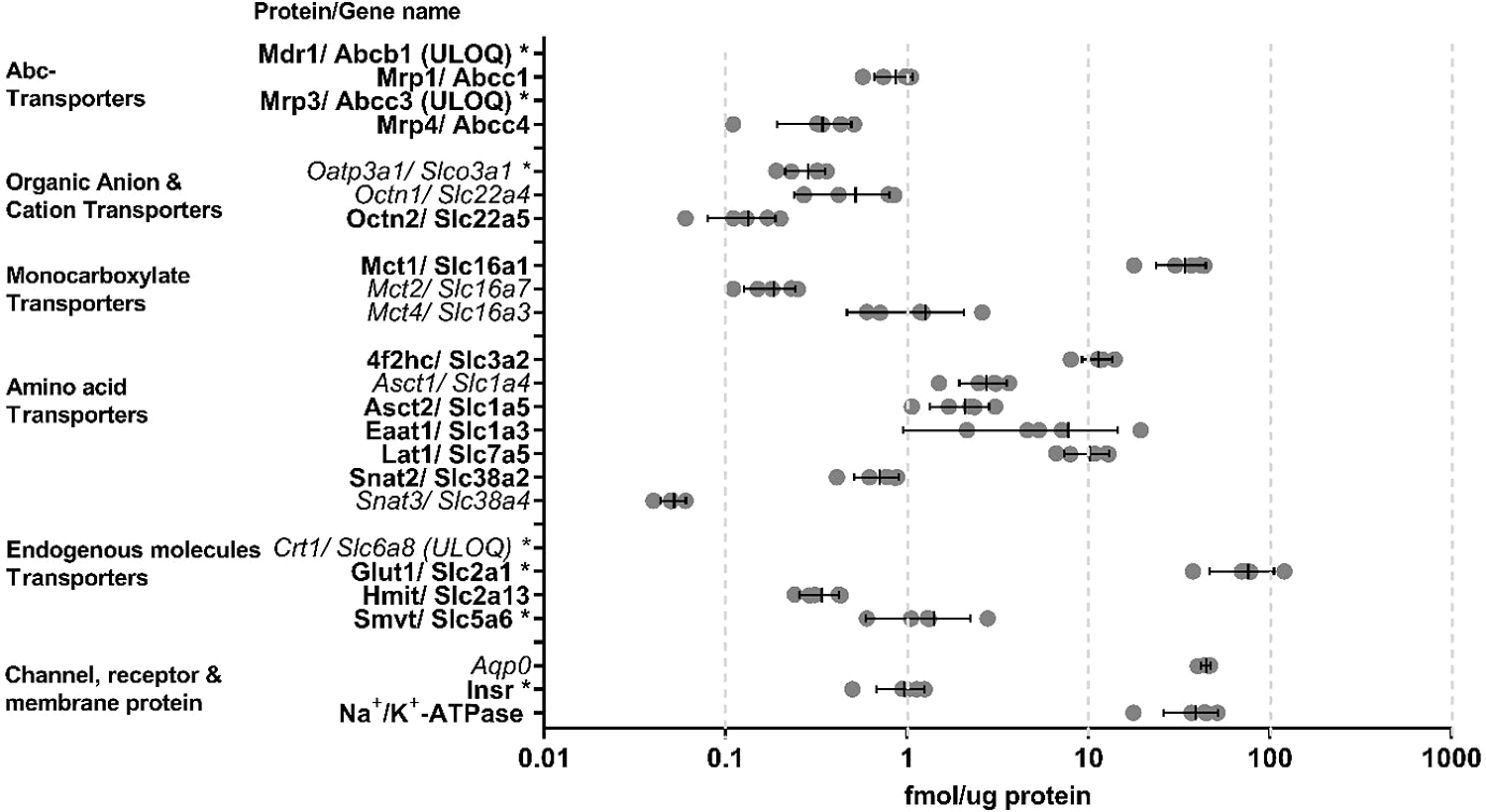
Protein expression levels in rabbit CE plasma membrane-rich fractions (*n* = 5). The symbols represent the mean expression level for each fraction. The two directional error bars with the middle line represents the mean value and standard deviation of the five fractions, respectively. Mdr1, Mrp3, and Crt1 are detected but ULOQ. Proteins detected by both targeted and untargeted approaches (Orbitrap and TimsTOF Pro) are marked in bold at the *y*-axis, whereas proteins detected only by the targeted approach are marked in italic. Proteins detected by only two transitions by the targeted approach is marked with an asterisk.

**Figure 2:**
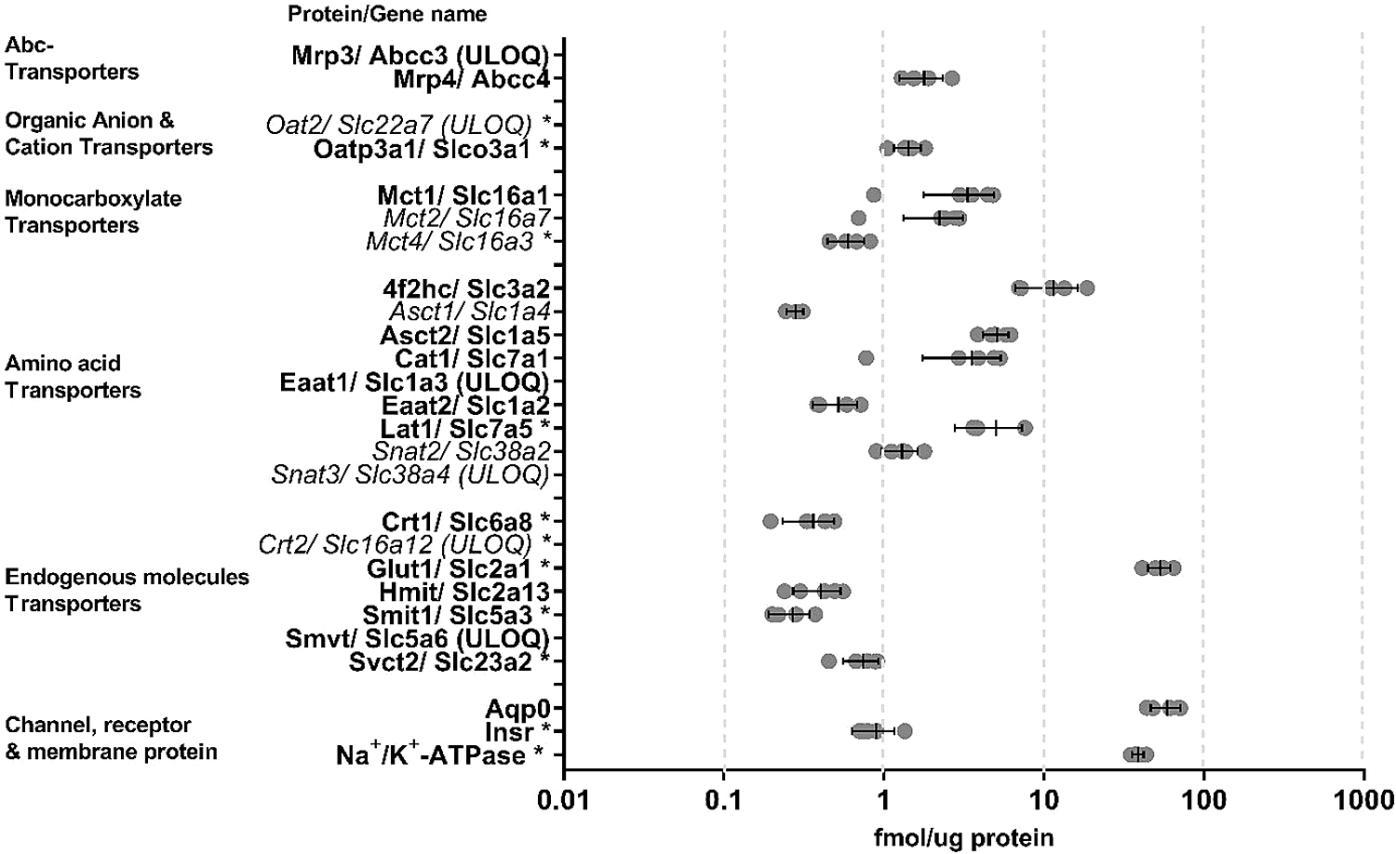
Protein expression levels in porcine CE plasma membrane-rich fractions (*n* = 3–5). The symbols represent the mean expression level in each fraction. The two directional error bars with the middle line represent the standard deviation and the mean value of the fractions, respectively. Mct2, Mct4, Oat2, Snat2, Snat3, and Asct1 are detected but under the limit of quantification (ULOQ). Proteins detected by both targeted and untargeted approaches are marked in bold at the *y*-axis, whereas proteins detected only by the targeted approach are marked in italic. Proteins detected by only two transitions by the targeted approach are marked with an asterisk. In the targeted approach, five plasma membrane-rich fractions were analyzed, but for Lat1 and Asct1 the expression levels were quantified with only 3 fractions, and for Eaat2, Crt1 and Smit1 only 4 fractions were used.

The expression of 48 and 58 proteins was studied in both plasma membrane-rich and total cell lysate fractions of CE from rabbit and porcine, respectively (Figures 1 and 2; Table 1). Forty-eight proteins were studied in both species, which included 8 ATP-binding cassette transporters (Abc), 37 solute carrier (Slc) transporters, aquaporin 0 (Aqp0), insulin receptor (Insr), and Na^+^/K^+^-ATPase. Five independent plasma membrane-rich and total cell lysate samples were analyzed for both animals together with a standard curve in triplicate for each animal. The heavy peptide sequences used in the quantitation of each animal-specific protein are shown in Supporting Information File 1.

**Table 1:** Proteins Not Detected or Under the Limit of Quantification (ULOQ) in Rabbit and Porcine Plasma Membrane-Rich Fractions^a^^,^^b^

protein/gene name	values of the limit of quantification (fmol/μg protein)	
	rabbit	porcine
Abca/Abca1	**	0.8
*Bcrp/Abcg2*	*0.04*	*0.04*
Bsep/Abcb11	0.01	0.2
*Crt1/Slc6a8*	0.2*	*quantified*
Crt2/Slc16a12	0.8	0.02*
*Eaat1/Slc1a3*	*quantified*	0.8*
Eaat2/Slc1a2	0.02	quantified
Eaat3/Slc1a1	**	0.04
Eaat4/Slc1a6	0.2	0.04
Mct5/Slc16a4	0.04	0.04
*Mdr1/Abcb1*	0.04*	*0.02*
*Mrp1/Abcc1*	*quantified*	*0.04*
Mrp2/Abcc2	0.04	0.02
*Mrp3/Abcc3*	0.2*	0.2*
*Mrp5/Abcc5*	*0.04*	*0.2*
Ntt4/Slc6a17	0.8	0.8
Oat1/Slc22a6	0.02	0.2
Oat2/Slc22a7	0.04	0.2*
Oat3/Slc22a8	0.04	0.2
Oat4/Slc22a11	**	0.2
Oatp1a2/Slco1a2	0.04	0.8
*Oatp1c1^YI/Slco1c1*	*0.8*	*0.8*
Oatp1c1^LY/Slco1c1	0.2	
Oatp2a1/Slco2a1	0.02	0.02
Oatp2b1/Slco2b1	0.2	0.01
Oatp4a1/Slco4a1	**	0.2
Oatp4c1/Slco4c1	0.2	0.04
Oatp6a1/Slco6a1	**	0.02
Oct1/Slc22a1	0.2	0.04
*Oct2/Slc22a2*	*0.2*	*0.2*
Oct3/Slc22a3	0.2	0.04
Octn1/Slc22a4	quantified	0.04
Octn2^FQ/Slc22a5		0.8
*Octn2^WL/*Slc22a5	*quantified*	*0.02*
OST-α /Slc51a	***	0.04
OST-β/ Slc51b	**	***
*Pept1^TL/Slc15a1*	*0.02*	*0.04*
Pept1^DS/Slc15a1		0.8
Pept2/Slc15a2	0.02	0.8
*Smit1/Slc5a3*	*0.02*	*quantified*
Smvt/Slc5a6	quantified	0.2*
*Snat3/Slc38a4*	*quantified*	0.2*
Snat5/Slc38a5	**	0.04
Svct1/Slc23a1	**	0.02

a The lower limit of quantification (LLOQ) was determined based on the criteria listed in Supplementary File 1. Heavy peptide sequences for rabbit and porcine proteins are mainly different except for proteins marked in italic.

b * Proteins were not studied in rabbits; ** Protein detected but ULOQ; *** No signal in LC-MS/MS; ^ Defines the two first amino acids of the peptide sequence.

For the rabbit proteins, 51 peptide sequences were used in the targeted approach, where Na^+^/K^+^-ATPase, Lat1, and Oatp1c1 had two peptide sequences. Of these peptide sequences, 23 sequences were selected using the *in silico* criteria (Supplementary File 1). For the porcine proteins, we used 62 peptide sequences in the targeted method, where Crt1, Mrp4, Octn2, and Pept2 had two peptide sequences. Among the porcine peptide sequences, 24 sequences were selected using the *in silico* criteria (Supplementary File 1).

Among rabbit plasma membrane fractions, 24 proteins of 48 were detected by targeted proteomics (Figure 1). Overall, 21 proteins were quantified, and three proteins, Mdr1, Mrp3, and Crt1, were under the limit of quantification (ULOQ). The lower limits of quantification (LLOQ) of rabbit proteins that were not detected or were ULOQ are shown in Table 1.

In the porcine plasma membrane-rich fractions, a total of 26 proteins of 58 were detected by targeted proteomics (Figure 2). Twenty proteins were quantified, and 6 proteins Mrp3, Oat2, Smvt, Snat3, Eaat1, and Crt2 were ULOQ. The LLOQ for porcine proteins that were not detected or were ULOQ is shown in Table 1.

A complete list of the studied proteins with their quantitative expression values for each plasma membrane-rich fraction and total cell lysate fraction for rabbit and porcine as well as the lower limit of detection (LLOD) and lower limit of quantification (LLOQ) for all heavy peptides used in the study are shown in Supporting Information File 1.

**Figure 3:**
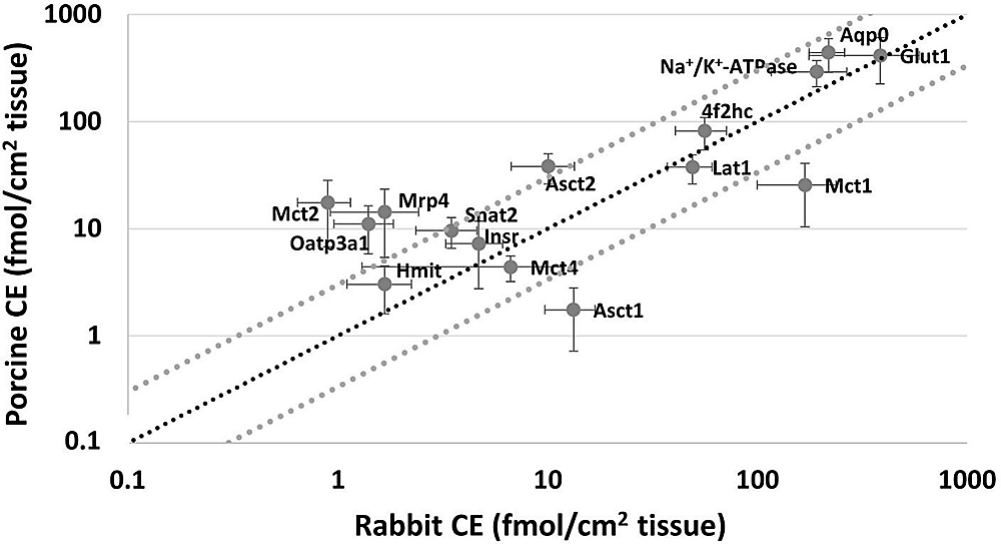
Protein expression (fmol/cm^2^; mean ± standard deviation) in the CE of porcine and rabbit. The figure shows all proteins that were successfully quantified in both animals. The black dotted line represents identical expression, and the gray dotted lines represent a 3-fold difference from identical expression. Transporters Mrp3, Eaat1, Crt1, and Snat3 were detected in both species, but expression was ULOQ in one or both animals.

A total of 19 proteins were detected in the CE of rabbit and porcine, including 1 Abc and 15 Slc transporters. Figure 3 illustrates the expression (fmol/cm^2^) of 15 proteins that were successfully quantified in both species. A complete list of converted values for all quantified proteins in rabbit and porcine plasma membrane-rich fractions and total cell lysate fractions are shown in Supplementary File 1. Most proteins in Figure 3 have equal or within 3-fold expression levels in both animals. However, Asct2, Mct2, Mrp4, and Oatp3a1 show a higher expression level in porcine CE, whereas Mct1 and Asct1 are expressed more in rabbit CE.

### Untargeted Global Proteomics of Rabbit and Porcine CE

Plasma membrane-rich samples of rabbit and porcine corneal epithelia were also analyzed by two different instruments and untargeted label-free quantification methods, i.e., DIA analysis by Orbitrap Q-Exactive (Thermo) and diaPASEF by timsTOF pro (Bruker). The untargeted proteomics was mainly used to select transporter proteins for the targeted proteomics study. Interestingly, 93% of the proteins identified by Orbitrap in porcine CE were also identified by timsTOF pro. Similarly, 85% of proteins identified by Orbitrap in rabbit CE were also identified by timsTOF pro.

Most of the proteins detected with the targeted method (24) in rabbit plasma membrane-rich fractions were also detected by the untargeted approaches (16) (Figure 1). However, Mct2, Mct4, Oatp3a1, Octn1, Snat3, Asct1, Aqp0, and Crt1 were only detected with the targeted approach, whereas Smit1 and Crt2 were detected only by the diaPASEF method. In addition to the 48 proteins studied in both species, the porcine epithelium showed expression of 10 extra proteins in the targeted approach. Of these 3 proteins, Abca, Svct2, and Cat1 were detected in the rabbit plasma membrane-rich fractions in the untargeted approaches.

The porcine proteins detected by the targeted approach (26) were also detected by the untargeted approaches (19) (Figure 2). However, Mct2, Mct4, Oat2, Snat2, Snat3, Asct1, and Crt2 were only detected with the targeted approach. Additionally, the untargeted diaPASEF method revealed more porcine proteins, e.g., Mdr1, Mrp1, Mrp5, Oatp2a1, Eaat4, and Abca1, which were not detected with the targeted method. Information on all rabbit and porcine proteins studied in the targeted approach and whether they were detected in the targeted and untargeted approaches are shown in Supplementary File 2.

The untargeted approach identified additional important Slc and Abc transporters, as well as receptors and enzymes. The results from the untargeted methods are shown in Supporting Information File 3. Based on the timsTOF pro global untargeted proteomics data, a Venn diagram and heatmaps were generated to illustrate the detection intensity and species differences of some selected transporters, receptors, and enzymes (Supplementary File 4). Enzymes such as N(alpha)-acetyltransferase (NAA15, NAA20, NAA25, NAA50, NAAA, NAT1), glutathione S-transferase (GSTA4,; GSTM3), UDP-glucuronyl transferase (UGT1, UGT2B13), carbonic anhydrase (CA1, CA2, CA5B, CA13), and tight-junction proteins (claudine (CLDN1, CLDN4, CLDN7, CLDN8, CLDN23), occludin (OCLN), and junctional adhesion molecule A (F11R)) were detected with the diaPASEF in both rabbit and porcine CE plasma membrane-rich fractions.

## Discussion

Transcorneal drug permeation is required in the topical treatment of anterior eye tissues. The corneal permeability is governed by both passive and/or active transport, but the impact of active transport in transcorneal permeability is still unclear. Herein, we present absolute quantitative protein expression data of drug and amino acid transporters in plasma membrane-rich fractions of rabbit and porcine CE. The quantitative expression data were supported by the untargeted global proteomic screening. The screening generated high-throughput data, which revealed the expression of many other proteins, such as receptors, enzymes, and tight-junction proteins that are important in understanding of transcorneal pharmacokinetics and physiological functions (Supplementary Files 3 and 4).

### Drug Transporters

The detected rabbit and porcine proteins were comparable to available literature data on qualitative protein expression (Supplementary File 2) with some exceptions. The protein expression profile for rabbit cornea is better known than that of the porcine cornea.^12,15^ Many clinical drugs, also in ocular therapy, are recognized as substrates or inhibitors of Abc and Slc transporters, such as multidrug resistance-associated proteins (Mrp), multidrug resistance protein (Mdr1), monocarboxylate transporter (Mct), organic anion and cation transporters (Oat, Oct), organic anion transporting polypeptide (Oatp), and peptide transporter (Pept).^14,55^

We included 8 Oatps (Supplementary Files 1 and 2) in our targeted approach, but only Oatp3a1 was detected and quantified in both rabbit (1.4 ± 0.4 fmol/cm^2^) and porcine (11.1 ± 5.3 fmol/cm^2^) corneal epithelia (Supplementary File 1). Additionally, Oatp2b1 was only detected by diaPASEF in porcine CE. Oatp3a1 was previously quantified in the porcine choroid plexus, brain capillary, and retinal pigment epithelium.^34^ With its multiple isoforms, the location of Oatp3a1 can vary from basolateral to apical expression.^56,57^ The cell-specific location in CE is still unknown, but in the choroid plexus, OATP3A1_v1 was located to the basolateral side and OATP3A1_V2 and _V3 to the apical side.^56,57^ Its physiological role is still unknown, but in a study by Pan et al.,^58^ OATP3A1 was upregulated as a response to cholestasis in the liver. They also showed the OATP3A1 as a bile acid efflux transporter.^58^ On the other hand, OATP3A1 has been shown to act as an uptake transporter for estrone-3-sulfate, prostaglandins, thyroxine, and vasopressin^57−59^ Hydrophilic bile acid ursodeoxycholic acid (UDCA) and its conjugate tauroursodeoxycholic acid (TUDCA) have shown neuroprotective properties in a range of *in vivo* retinal diseases models^60,61^ and alleviation in cataract formation in induced rat models.^62,63^ The role of OATP3A1 in bile acid homeostasis in the eye and in the pathogenesis of ocular disease requires further investigations. It has been suggested to be a potential pharmacological target for drug delivery to the brain;^56,59^ however, due to its varying cell location in different organs and bidirectional transporter properties, its role as a potential drug delivery route in the CE requires further investigation.

The efflux transporter Mrp4 was quantified in both rabbit (1.7 ± 0.8 fmol/cm^2^) and porcine (14.3 ± 8.9 fmol/cm^2^) CE (Supplementary File 1), showing Mrp4 expression for the first time in rabbit. Previously, Verstraelen and Reichl^64^ did not detect the transporter expression nor function with the use of adefovir uptake in rabbit cornea. In the same study, both the expression and functionality of Mrp2 and Mrp5 were detected, but these transporters were not detected in this study. Similarly, Mct5 and Pept1 were not detected in rabbit CE, in contrast to previous qualitative data on expression.^65,66^ Some of these contradictory results may be explained by the use of unspecific antibodies in previous qualitative studies. In porcine CE, drug transporters Mdr1 and Mrp1 have not been detected previously.^64,67^ This was supported by the targeted approach, but untargeted proteomics showed these transporters. Porcine Mrp5, previously identified,^64^ was detected only with the untargeted approach.

Peptide transporter-mediated drug delivery has been studied for improving transcorneal delivery of prodrugs, such as ganciclovir and acyclovir.^68−71^ These studies have shown peptide-associated transcorneal delivery of antiviral prodrugs in excised rabbit cornea.^69,71^ However, we demonstrate that neither Pept1 nor Pept2 were detected, or they were below the LLOD in rabbit CE. The LLOD for these two proteins in rabbits was indeed very low (0.004 fmol/μg protein), suggesting that if they are expressed, their abundance would likely be extremely low. In the human cornea, the peptide transporters have so far been detected to our knowledge only at the mRNA level.^72,73^

### Amino Acid Transporters

The function and viability of the CE require active uptake of nutrients, growth factors, and amino acids from the tear fluid and the corneal stroma.^74^ The expression and functionality of amino acid transporters in the cornea have been studied less than those of drug transporters. Several amino acid transporters were detected by the targeted approach in porcine CE: large neutral amino acid transporter 1 (Lat1), neutral amino acid transport system Asct1 and 2 (alanine, serine, cysteine, threonine) transporter, excitatory amino acid transporter 1 and 2 (Eaat1, 2), amino acid transporter heavy chain (4f2hc), sodium-coupled neutral amino acid transporter A2 and A3 (Snat2 and Snat3), and cationic amino acid transporter 1 (Cat1). In rabbit CE, the same transporters were quantified, except Eaat2 (not detected) and Cat1 (detected only with an untargeted approach).

Lat1 has some potential in CNS drug delivery.^75,76^ It was quantified with new peptide sequences in rabbit and porcine CE. In the rabbit, Lat1 quantification was done with two peptides: (1) transmembrane domain peptide (VQDAFAAAK) previously used in mouse and human studies^32,77,78^ and (2) new extracellular domain peptide (EAEAEGEGVALQR). The extracellular domain peptide showed 8.5-fold higher expression (48.9 ± 11.8 fmol/cm^2^) than the transmembrane domain peptide (5.8 ± 1.7 fmol/cm^2^). Porcine Lat1 was also quantified with a new extracellular domain peptide (PAEGEGVTLQR) at a level of 37.6 ± 11.5 fmol/cm^2^ (Supplementary File 1). The differences can be probably explained by insufficient trypsin digestion of the transmembrane domain. In future studies, the new peptide sequences should be used for Lat1 quantification. The visual location of the peptide sequence in the rabbit and porcine Lat1 can be seen in the Supplementary File 1. The Lat1 data are supported by studies on mRNA expression and functionality in rabbit cornea.^79^

The targeted proteomics results of Asct1 (*R*: 13.3 ± 3.6 and *P*: 1.8 ± 1.0 fmol/cm^2^) (Supplementary File 1) are in agreement with RNA expression and functionality data for rabbit CE, where l-alanine transport was studied.^80^ Moreover, 4F2hc and EAAT1 have been detected in the human cornea before,^74^ but now we present their quantitative expression in the CE for the first time in both rabbits (55.9 ± 15.3 and 40.9 ± 43.7 fmol/cm^2^, respectively) and porcine (81.7 ± 27.3 and ULOQ fmol/cm^2^, respectively). Similarly, Snat2 and 3 were detected for the first time in the CE of rabbit (3.5 ± 1.1 and 0.3 ± 0.1 fmol/cm^2^, respectively) and porcine (9.6 ± 3.1 and ULOQ fmol/cm^2^, respectively). Previously, its expression and function have been studied in retinal Müller cells and in the inner blood–retinal barrier.^81,82^ Cat1 was quantified in porcine CE (26.1 ± 12.8 fmol/cm^2^) and detected by the untargeted approaches in rabbit CE. Previously, human mRNA expression was studied in pathological ocular disorders.^83^ Our data support the potential of amino acid transporters as targets for amino acid-based drug and prodrug delivery.

### Endogenous Molecule Transporters

In porcine CE, myoinositol (Smit1 and Hmit), vitamin C (Svct2), creatine (Crt1, 2), glucose (Glut1), and multivitamin (Smvt) transporters, as well as aquaporin (Aqp0) were detected by the targeted approach in porcine CE. In rabbit CE, the profile for endogenous molecule transporters varied, and Hmit, Glut1, Crt1, Aqp0, and Smvt were all detected in the targeted approach. Additionally, Svct2 was detected via the untargeted proteomic approach.

Aquaporins (Aqp) are water channels involved in the fluid transport across cell membranes and also present in many important barriers of the eye.^84^ Strong evidence shows that Aqp3 and Aqp5 are expressed in the CE.^85^ Aquaporins have also been investigated as potential targets in the treatment of age-related eye disorders such as glaucoma.^86^ The high expression in the CE (*R*: 218.2 ± 41.8 and *P*: 440.6 ± 152.9 fmol/cm^2^) supports the potential of using the aquaporins as therapy targets. Multivitamin transporter, Smvt is primarily responsible for the translocation of vitamins and other important cofactors such as biotin.^87^ The RNA expression of Smvt and biotin uptake was previously shown in rabbit cornea.^88^ Similarly, biotinylated-ganciclovir was recognized by Smvt in the retinal pigment epithelium and rabbit retina.^89^ The literature data on biotin uptake support the potential of Smvt-mediated transcorneal delivery, regardless of its relatively low expression in rabbits (7.3 ± 5.6 fmol/cm^2^) and ULOQ in porcine.

Vitamin C transporter Svct2 was quantified in the porcine CE (5.7 ± 2.7 fmol/cm^2^) and detected in the untargeted approach in rabbit CE (not included in the targeted approach). These findings are in agreement with previously published data in which rabbit corneal epithelial cells showed RNA expression of Svct2 and l-ascorbic acid uptake was shown to be Svct2 carrier-mediated.^90^ Vitamin C acts as an antioxidant by scavenging reactive oxygen species, absorbing UV radiation, and protecting the cornea and other ocular tissues from light-induced damage.^90^ The vitamin C concentrations in the aqueous and vitreous humor are much higher than in the plasma,^91,92^ and decreased levels of vitamin C in the lens have been identified with cataract severity.^93^ Svct2-mediated transcorneal delivery might serve as a potential route for intraocular delivery of vitamin C.

### Species Differences in Protein Expression

The quantitative protein expression was converted to fmol/cm^2^ of CE, to better compare the protein expression in the two animal models and to consider transporter protein function in the tissues. Transporter proteins such as 4f2hc, Asct1, Asct2, Hmit, Lat1, Mct1, Mct2, Mct4, Mrp4, Oatp3a1, and Snat2 were all quantified in the CE of both rabbit and porcine. The comparison of the two species showed a slightly higher expression (over 3-fold) of proteins in the porcine compared with the rabbit CE (Figure 3). This difference might be explained by the epithelial thickness and number of epithelial cell layers in the CE of porcine and rabbit. The thickness of porcine CE is 50–70 μm and it is 6–9 cell layer thick, whereas in the rabbit CE is 30–40 μm in thickness and contains 5–7 cell layers.^94^ In this context, it is important to highlight that our calculations only considered the surface area of the cornea and not the CE thickness or number of cell layers.

Rabbit is extensively used as an animal model in preclinical ocular studies.^9,95,96^ The isolated rabbit and porcine cornea have been used in a variety of drug permeability, delivery, and transporter activity studies.^10,27,28,31,64,67,97−100^ However, their suitability and translational ability to the human cornea, in terms of transporter expression profile, are not known. Compared with the current data on transporter expression in humans, there are some species differences observed in both rabbit and porcine corneas (Supporting Information Files 2 and 4). BCRP is a clinically important efflux transporter that is expressed in the human cornea.^101,102^ However, this transporter is not detected in rabbit or porcine corneas (confirmed by targeted and untargeted approaches). There is no information on MDR1 expression in humans; however, it is detected but ULOQ in rabbit CE, as well as in porcine CE in the untargeted approach. Among the MRP efflux transporters, there are additional variations. MRP1–5 were reported to be expressed in the human cornea.^101−103^ For example, MRP4 has been shown to be clinically relevant in the permeability of latanoprost, in the treatment of glaucoma.^104^ On the other hand, our data indicate that Mrp2 and Mrp5 are not found, whereas Mrp1, 3, and 4 are quantified or ULOQ in rabbit cornea (Mrp1: 4.3 ± 1.4; Mrp3: ULOQ; Mrp4: 1.7 ± 0.8 fmol/cm^2^, respectively). In the porcine Mrp data, there is more variation; for example, Mrp1 and Mrp5 are only detected in the untargeted approaches, whereas the rest of the Mrps (Mrp3: ULOQ; Mrp4:14.3 ± 8.9 fmol/cm^2^) have a similar profile as in the rabbit CE. Among the SLC transporters, the information is scarce in humans. OAT2, OATP2A1, OATP2B1, and OCT3 are all detected by immunohistochemical staining in the human cornea,^102,105^ but none of these transporters were detected in the rabbit cornea by the targeted nor untargeted approaches. Oat2 (ULOQ) and Oatp2a1 were detected in porcine CE by the targeted and untargeted approaches, respectively. The lack of detection may also partly be explained by the fact that Oatp2a1 has been found to localize in the lysosomes.^106^ From the anatomical aspect, the human CE thickness and number of cell layers is the same as for rabbit,^94^ but the surface area of the rabbit cornea is 1.6 times larger than human CE,^51^ making the rabbit CE more exposed to substances. Based on our study, the expression profiles of CE plasma membrane-rich transporters of porcine and rabbit had similarities. Quantitative expression data are, however, needed for the human CE for systematic comparison of transporter expression and expression levels.

### Targeted and Global Proteomics

The targeted approach of protein quantification offers enhanced sensitivity at subfemtomole range. This is achieved by carefully selecting one or two proteotypic peptides per protein and calculating the absolute protein quantity based on the ratio between the native and isotopically labeled standard peptides. As a result, the analysis by triple quadrupole MS with the SRM/MRM mode enables us to have sufficient sensitivity by enhancing the signal-to-noise ratios. However, these methods are limited by the number of coeluting peptides/transitions that can be followed per instrument duty cycle. In this study, we utilized the new dynamic MRM approach to maximize the number of peptides that can be measured simultaneously (acquisition method details, Supplementary File 1). The MRM methods allowed us to quantify all of these proteins from one single experiment/run without compromising the signal quality. These MRMs can even be applied to quantify up to 120–200 peptides/proteins in a single LC gradient method. On the other hand, the untargeted approach offers a high-throughput relative protein quantification strategy. For instance, the DIA method of Orbitrap Q-Exactive enabled the relative quantification of 3162 and 4103 rabbit and porcine plasma membrane proteins in a 90 min single run, respectively (Supplementary File 3). Similarly, the diaPASEF of the timsTOF Pro instrument enabled the relative quantification of 4851 and 5899 rabbit and porcine plasma membrane proteins in 20 min single run, respectively (Supplementary File 3). The targeted protein quantification approach was able, for instance, to detect some transporters better than that in the untargeted global proteomics method. On the other hand, the global proteomics approach detected a few proteins that were not detected in the targeted proteomic approach. This might be due to the capability of the global proteomic approach to detect some miscleaved or modified peptides such as peptides containing methionine oxidation, as the targeted approach is based on the detection of highly reliable and stable peptides. The global proteomics approach is a valuable tool in screening cells for proteins and in supporting the selection of unique peptides for the targeted approach. In this study, we only had two plasma membrane-rich replicate samples for the porcine CE and only one plasma membrane-rich sample of rabbit CE for the untargeted approach. Therefore, the global proteomic approach served as a valuable addition to back up the findings of the targeted proteomics data.

### Future Aspects

Information on ocular barriers and their transporters are key factors in the successful design of new ocular drugs, pharmacokinetic modeling, and understanding ocular diseases. Ocular drug development involves tissues and cells from rabbits, but human translation is limited by a poor understanding of active transport in the cornea of these two species. The data presented herein add important information to the public domain. Targeted and global proteomics provide valuable high-throughput methods to screen a large range of proteins in a short time compared with the qualitative methods that require more time. The presented large dataset provides quantitative protein expression data that may be utilized in building physiologically based pharmacokinetic models.

The CE (basal and superficial layers) and cell-specific (basolateral and apical) location of transport proteins are very important information for predicting and understanding the impact of transporter activity on drug pharmacokinetics. Immunohistochemical staining has been used to distinguish the protein location on the multilayered CE, but the information is still sparse.^12^ The direction of drug transport across corneal epithelial cells is regulated by the polarized transporter protein expression in the apical and/or basolateral plasma membranes. Most likely the expression of some transporters in the CE is polarized, which leads to the transport of substances toward tear fluid or aqueous humor. Immunocytochemical analysis with antibodies and protein quantification of fractionated plasma membranes with cell location-specific marker proteins are methods available for studying the polarization of transporters.^33,107,108^ Although sample availability of human cornea is limited and functional characterization of active transport *in vivo* is not possible, transporter protein quantification would still provide useful information for the understanding of transporter functional activity.^39^ These steps, determining cellular localization and quantifying transporters in the human CE are the next steps to be taken to better understand the value and limitations of the animal models. The large datasets also provide reference data on protein expression levels in healthy animals. The etymology of many ocular diseases is complex, and to understand them better, we need to look at protein expression levels in disease states to better understand disease and potential drug target sites. In the future, it is also important to integrate polarized transporter expression levels and functionality in pharmacokinetic models. These models and tools speed up the development of drugs but also the reduction and replacement of *in vivo* animal experiments.

In conclusion, we were able to absolutely quantify 21 and 20 proteins, mainly Abc and Slc transporters in rabbit and porcine CE, respectively. Among these, 15 proteins were quantified in both species, showing some similarities and variations in the expression. The untargeted proteomics screening data supported the targeted data in most cases.

## Supporting Information Available

Supplementary File 1: Targeted proteomics information and data (XLSX)
Supplementary File 2: Literature data on protein expression in human, rabbit, and porcine cornea, as well as proteins detected in the targeted and untargeted global proteomics of rabbit and porcine CE (PDF)
Supplementary File 3: Untargeted global proteomics data (XLSX)
Supplementary File 4: Heatmaps and Venn diagrams of selected data from the untargeted proteomics, timsTOF pro analysis (XLSX)

## Abbreviations

4f2hc: amino acid transporter heavy chain
Abc: ATP-binding cassette transporters
Abca: ATP-binding cassette, subfamily A
Aqp0: aquaporin 0
Asct: alanine/serine/cysteine/threonine transporter
Bcrp: breast cancer resistance protein
Bsep: bile salt export pump
CE: corneal epithelium
Cat: cationic amino acid transporter
Crt: creatine transporter
DDA: data-dependent acquisition
DIA: data-independent acquisition
diaPASEF: parallel accumulation–serial fragmentation combined with data-independent acquisition
MRM: multiplexed multiple reaction monitoring
Eaat: excitatory amino acid transporter
Glut: glucose transporter
Hmit: H(+)-myoinositol cotransporter
Insr: insulin receptor
Lat: L-type amino acid transporter
LLOD: lower limit of detection
LLOQ: lower limit of quantification
Mct: monocarboxylate transporter
Mdr: multidrug resistance protein, also known as P-glycoprotein
Mrp: multidrug resistance-associated protein
Na^+^/K^+^ - ATPase: sodium/potassium-transporting ATPase
Ntt: neurotransmitter transporter
Oat: organic anion transporter
Oatp: organic anion transporting polypeptide
Oct: organic cation transporter
Octn: organic cation transporters novel
Ost-a and b: organic solute transporter, alpha and beta subunit
Pept: peptide transporter
Slc: solute carrier
Smit: sodium/myoinositol cotransporter
Smvt: sodium-dependent multivitamin transporter
Snat: sodium-coupled neutral amino acid transporter
Svct: sodium-dependent vitamin C transporter
ULOD: under limit of detection
ULOQ: under limit of quantification

## References

[ref1] Urtti A. Challenges and obstacles of ocular pharmacokinetics and drug delivery. Adv. Drug Deliv Rev. 2006, 58, 1131–1135. 10.1016/j.addr.2006.07.027. 17097758

[ref2] Subrizi A.; del Amo E. M.; Korzhikov-Vlakh V.; Tennikova T.; Ruponen M.; Urtti A. Design principles of ocular drug delivery systems: Importance of drug payload, release rate, and material properties. Drug Discov Today 2019, 24, 1446–1457. 10.1016/j.drudis.2019.02.001. 30738982

[ref3] Ahmed I.; Patton T. F. Importance of the noncorneal absorption route in topical ophthalmic drug delivery. Invest. Ophthalmol. Vis. Sci. 1985, 26, 584–587. 3884542

[ref4] Ahmed I.; Patton T. F. Disposition of timolol and inulin in the rabbit eye following corneal versus non-corneal absorption. Int. J. Pharm. 1987, 38, 9–21. 10.1016/0378-5173(87)90092-5.

[ref5] Doane M. G.; Jensen A. D.; Dohlman C. H. Penetration routes of topically applied eye medications. Am. J. Ophthalmol 1978, 85, 383–386. 10.1016/S0002-9394(14)77735-9. 655217

[ref6] Chrai S. S.; Robinson J. R. Corneal permeation of topical pilocarpine nitrate in the rabbit. Am. J. Ophthalmol 1974, 77, 735–739. 10.1016/0002-9394(74)90541-8. 4823783

[ref7] Fayyaz A.; Ranta V. P.; Toropainen E.; Vellonen K. S.; Valtari A.; Puranen J.; Ruponen M.; Gardner I.; Urtti A.; Jamei M.; del Amo E. M. Topical ocular pharmacokinetics and bioavailability for a cocktail of atenolol, timolol and betaxolol in rabbits. European Journal of Pharmaceutical Sciences 2020, 155, 105553 10.1016/j.ejps.2020.105553. 32946960

[ref8] Naageshwaran V.; Ranta V.; Gum G.; Bhoopathy S.; Urtti A.; del Amo E. M. Comprehensive ocular and systemic pharmacokinetics of brinzolamide in rabbits after intracameral, topical, and intravenous administration. J. Pharm. Sci. 2021, 110, 529–535. 10.1016/j.xphs.2020.09.051. 33035542

[ref9] Naageshwaran V.; Ranta V. P.; Toropainen E.; Tuomainen M.; Gum G.; Xie E.; Bhoopathy S.; Urtti A.; del Amo E. M. Topical pharmacokinetics of dexamethasone suspensions in the rabbit eye: Bioavailability comparison. Int. J. Pharm. 2022, 615, 121515 10.1016/j.ijpharm.2022.121515. 35091006

[ref10] Ramsay E.; del Amo E. M.; Toropainen E.; Tengvall-Unadike U.; Ranta V. P.; Urtti A.; Ruponen M. Corneal and conjunctival drug permeability: Systematic comparison and pharmacokinetic impact in the eye. European Journal of Pharmaceutical Sciences 2018, 119, 83–89. 10.1016/j.ejps.2018.03.034. 29625211

[ref11] Urtti A.; Pipkin J. D.; Rork G.; Sendo T.; Finne U.; Repta A. J. Controlled drug delivery devices for experimental ocular studies with timolol 2. Ocular and systemic absorption in rabbits, Int. J. Pharm. 1990, 61, 241–249. 10.1016/0378-5173(90)90215-P.

[ref12] Vellonen K. S.; Hellinen L.; Mannermaa E.; Ruponen M.; Urtti A.; Kidron H. Expression, activity and pharmacokinetic impact of ocular transporters. Adv. Drug Deliv Rev. 2018, 126, 3–22. 10.1016/j.addr.2017.12.009. 29248478

[ref13] Kidron H.; Vellonen K. S.; del Amo E. M.; Tissari A.; Urtti A. Prediction of the corneal permeability of drug-like compounds. Pharm. Res. 2010, 27, 1398–1407. 10.1007/s11095-010-0132-8. 20387098

[ref14] Giacomini K. M.; et al. Membrane transporters in drug development. Nat. Rev. Drug Discov. 2010, 9, 215–236. 10.1038/nrd3028. 20190787 PMC3326076

[ref15] Mannermaa E.; Vellonen K. S.; Urtti A. Drug transport in corneal epithelium and blood-retina barrier: Emerging role of transporters in ocular pharmacokinetics. Adv. Drug Deliv Rev. 2006, 58, 1136–1163. 10.1016/j.addr.2006.07.024. 17081648

[ref16] Savinainen A.; Prusakiewicz J. J.; Oswald J.; Spencer E.; Lou Z.; Cohen M. L.; Rashidzadeh H.; Josiah S. Pharmacokinetics and intraocular pressure–lowering activity of TAK-639, a novel C-type natriuretic peptide analog, in rabbit, dog, and monkey. Exp. Eye Res. 2019, 189, 107836 10.1016/j.exer.2019.107836. 31626797

[ref17] Proksch J. W.; Lowe E. R.; Ward K. W. Ocular pharmacokinetics of mapracorat, a novel, selective glucocorticoid receptor agonist, in rabbits and monkeys. Drug Metab. Dispos. 2011, 39, 1181–1187. 10.1124/dmd.111.039099. 21441467

[ref18] Edelhauser H. F.; Maren T. H. Permeability of Human Cornea and Sclera to Sulfonamide Carbonic Anhydrase Inhibitors. Archives of Ophthalmology 1988, 106, 1110–1115. 10.1001/archopht.1988.01060140266039. 3401140

[ref19] Van Der Bijl P.; Engelbrecht A. H.; Van Eyk A. D.; Meyer D. Comparative Permeability of Human and Rabbit Corneas to Cyclosporin and Tritiated Water. Journal of Ocular Pharmacology and Therapeutics 2002, 18, 419–427. 10.1089/10807680260362704. 12419093

[ref20] Van Eyk A. D.; Van Der Bijl P.; Meyer D. In vitro diffusion of the immunosuppressant tacrolimus through human and rabbit corneas. Journal of Ocular Pharmacology and Therapeutics 2007, 23, 146–151. 10.1089/jop.2006.0105. 17444803

[ref21] Hämäläinen K. M.; Kananen K.; Auriola S.; Kontturi K.; Urtti A. Characterization of paracellular and aqueous penetration routes in cornea, conjunctiva, and sclera. Invest. Ophthalmol. Vis. Sci. 1997, 38, 627–634. 9071216

[ref22] Prausnitz M. R.; Noonan J. S. Permeability of cornea, sclera, and conjunctiva: a literature analysis for drug delivery to the eye. J. Pharm. Sci. 1998, 87, 1479–1488. 10.1021/js9802594. 10189253

[ref23] Wang W.; Sasaki H.; Chien D.-S.; Lee V. H. L. Lipophilicity influence on conjunctival drug penetration in the pigmented rabbit: a comparison with corneal penetration. Curr. Eye Res. 1991, 10, 571–579. 10.3109/02713689109001766. 1680041

[ref24] Loch C.; Zakelj S.; Kristl A.; Nagel S.; Guthoff R.; Weitschies W.; Seidlitz A. Determination of permeability coefficients of ophthalmic drugs through different layers of porcine, rabbit and bovine eyes. European Journal of Pharmaceutical Sciences 2012, 47, 131–138. 10.1016/j.ejps.2012.05.007. 22659372

[ref25] Hammid A.; Fallon J. K.; Lassila T.; Salluce G.; Smith P. C.; Tolonen A.; Sauer A.; Urtti A.; Honkakoski P. Carboxylesterase Activities and Protein Expression in Rabbit and Pig Ocular Tissues. Mol. Pharmaceutics 2021, 18, 1305–1316. 10.1021/acs.molpharmaceut.0c01154. PMC8023712 33595329

[ref26] Ahmed I.; Gokhale R. D.; Shah M. V.; Patton T. F. Physicochemical determinants of drug diffusion across the conjunctiva, sclera, and cornea. J. Pharm. Sci. 1987, 76, 583–586. 10.1002/jps.2600760802. 11002815

[ref27] Dey S.; Gunda S.; Mitra A. K. Pharmacokinetics of erythromycin in rabbit corneas after single-dose infusion: Role of P-glycoprotein as a barrier to in vivo ocular drug absorption. Journal of Pharmacology and Experimental Therapeutics 2004, 311, 246–255. 10.1124/jpet.104.069583. 15175422

[ref28] Karla P. K.; Pal D.; Quinn T.; Mitra A. K. Molecular evidence and functional expression of a novel drug efflux pump (ABCC2) in human corneal epithelium and rabbit cornea and its role in ocular drug efflux. Int. J. Pharm. 2007, 336, 12–21. 10.1016/j.ijpharm.2006.11.031. 17156953 PMC1995119

[ref29] Karla P. K.; Quinn T. L.; Herndon B. L.; Thomas P.; Pal D.; Mitra A. Expression of multidrug resistance associated protein 5 (MRP5) on cornea and its role in drug efflux. Journal of Ocular Pharmacology and Therapeutics 2009, 25, 121–132. 10.1089/jop.2008.0084. 19323627 PMC2963904

[ref30] Hariharan S.; Gunda S.; Mishra G. P.; Pal D.; Mitra A. K. Enhanced corneal absorption of erythromycin by modulating P-glycoprotein and MRP mediated efflux with corticosteroids. Pharm. Res. 2009, 26, 1270–1282. 10.1007/s11095-008-9741-x. 18958406 PMC4516224

[ref31] Hariharan S.; Minocha M.; Mishra G. P.; Pal D.; Krishna R.; Mitra A. K. Interaction of ocular hypotensive agents (PGF2 analogs - Bimatoprost, latanoprost, and travoprost) with MDR efflux pumps on the rabbit cornea. Journal of Ocular Pharmacology and Therapeutics 2009, 25, 487–497. 10.1089/jop.2009.0049. 20028257 PMC3096535

[ref32] Pelkonen L.; Sato K.; Reinisalo M.; Kidron H.; Tachikawa M.; Watanabe M.; Uchida Y.; Urtti A.; Terasaki T. LC-MS/MS Based Quantitation of ABC and SLC Transporter Proteins in Plasma Membranes of Cultured Primary Human Retinal Pigment Epithelium Cells and Immortalized ARPE19 Cell Line. Mol. Pharmaceutics 2017, 14, 605–613. 10.1021/acs.molpharmaceut.6b00782. 28112518

[ref33] Hellinen L.; Sato K.; Reinisalo M.; Kidron H.; Rilla K.; Tachikawa M.; Uchida Y.; Terasaki T.; Urtti A. Quantitative protein expression in the human retinal pigment epithelium: comparison between apical and basolateral plasma membranes with emphasis on transporters. Investigative Ophthalmology & Visual Scienceisual 2019, 60, 5022–5034. 10.1167/iovs.19-27328. 31791063

[ref34] Zhang Z.; Uchida Y.; Hirano S.; Ando D.; Kubo Y.; Auriola S.; Akanuma S. I.; Hosoya K. I.; Urtti A.; Terasaki T.; Tachikawa M. Inner Blood-Retinal Barrier Dominantly Expresses Breast Cancer Resistance Protein: Comparative Quantitative Targeted Absolute Proteomics Study of CNS Barriers in Pig. Mol. Pharmaceutics 2017, 14, 3729–3738. 10.1021/acs.molpharmaceut.7b00493. 28954515

[ref35] del Amo E. M.; Vellonen K. S.; Urtti A.; Terasaki T.; Hammid A.; Honkakoski P.; Auriola S. Mass spectrometry in ocular drug research. Mass Spectrom Rev. 2023, 1–32. 10.1002/mas.21861. PMC12706722 37530668

[ref36] HammidA.; FallonJ. K.; LassilaT.; VieiroP.; BallaA.; GonzalezF.; UrttiA.; SmithP. C.; TolonenA.; HonkakoskiP. Activity and Expression of Carboxylesterases and Arylacetamide Deacetylase in Human Ocular Tissues. Drug Metab. Dispos. 2022, 50, 1483–1492. 10.1124/dmd.122.000993.36195336

[ref37] Hammid A.; Fallon J. K.; Vellonen K. S.; Lassila T.; Reinisalo M.; Urtti A.; Gonzalez F.; Tolonen A.; Smith P. C.; Honkakoski P. Aldehyde oxidase 1 activity and protein expression in human, rabbit, and pig ocular tissues. European Journal of Pharmaceutical Sciences 2023, 191, 106603 10.1016/j.ejps.2023.106603. 37827455

[ref38] Kumar V.; Prasad B.; Patilea G.; Gupta A.; Salphati L.; Evers R.; Hop C. E. C. A.; Unadkat J. D. Quantitative transporter proteomics by liquid chromatography with tandem mass spectrometry: Addressing methodologic issues of plasma membrane isolation and expression-activity relationship. Drug Metab. Dispos. 2015, 43, 284–288. 10.1124/dmd.114.061614. 25488931

[ref39] Sakamoto A.; Suzuki S.; Matsumaru T.; Yamamura N.; Uchida Y.; Tachikawa M.; Terasaki T. Correlation of Organic Cation/Carnitine Transporter 1 and Multidrug Resistance-Associated Protein 1 Transport Activities with Protein Expression Levels in Primary Cultured Human Tracheal, Bronchial, and Alveolar Epithelial Cells. J. Pharm. Sci. 2016, 105, 876–883. 10.1002/jps.24661. 26429295

[ref40] Kamiie J.; Ohtsuki S.; Iwase R.; Ohmine K.; Katsukura Y.; Yanai K.; Sekine Y.; Uchida Y.; Ito S.; Terasaki T. Quantitative atlas of membrane transporter proteins: Development and application of a highly sensitive simultaneous LC/MS/MS method combined with novel in-silico peptide selection criteria. Pharm. Res. 2008, 25, 1469–1483. 10.1007/s11095-008-9532-4. 18219561

[ref41] Uchida Y.; Tachikawa M.; Obuchi W.; Hoshi Y.; Tomioka Y.; Ohtsuki S.; Terasaki T. A study protocol for quantitative targeted absolute proteomics (QTAP) by LC-MS/MS: Application for inter-strain differences in protein expression levels of transporters, receptors, claudin-5, and marker proteins at the blood-brain barrier in ddY, FVB, and. Fluids Barriers CNS 2013, 10, 1–22. 10.1186/2045-8118-10-21. 23758935 PMC3691662

[ref42] Montaser A. B.; Kuiri J.; Natunen T.; Hruška P.; Potěšil D.; Auriola S.; Hiltunen M.; Terasaki T.; Lehtonen M.; Jalkanen A.; Huttunen K. M. Enhanced drug delivery by a prodrug approach effectively relieves neuroinflammation in mice. Life Sci. 2022, 310, 121088 10.1016/j.lfs.2022.121088. 36257461

[ref43] Mikulášek K.; Konečná H.; Potěšil D.; Holánková R.; Havliš J.; Zdráhal Z. SP3 Protocol for Proteomic Plant Sample Preparation Prior LC-MS/MS. Front Plant Sci. 2021, 12, 635550 10.3389/fpls.2021.635550. 33777071 PMC7988192

[ref44] Wisniewski J. R. Filter Aided Sample Preparation e A tutorial Jacek. Anal. Chim. Acta 2019, 1090, 23–30. 10.1016/j.aca.2019.08.032. 31655642

[ref45] Hugele A.; Löffler S.; Molina B. H.; Guillon M.; Montaser A. B.; Auriola S.; Huttunen K. M. Aminopeptidase B can bioconvert L-type amino acid transporter 1 (LAT1)-utilizing amide prodrugs in the brain. Front Pharmacol 2022, 13, 1–12. 10.3389/fphar.2022.1034964. PMC9631218 36339537

[ref46] Meier F.; Brunner A. D.; Koch S.; Koch H.; Lubeck M.; Krause M.; Goedecke N.; Decker J.; Kosinski T.; Park M. A.; Bache N.; Hoerning O.; Cox J.; Räther O.; Mann M. Online parallel accumulation–serial fragmentation (PASEF) with a novel trapped ion mobility mass spectrometer. Mol. Cell. Proteomics 2018, 17, 2534–2545. 10.1074/mcp.TIR118.000900. 30385480 PMC6283298

[ref47] Meier F.; Brunner A. D.; Frank M.; Ha A.; Bludau I.; Voytik E.; Kaspar-Schoenefeld S.; Lubeck M.; Raether O.; Bache N.; Aebersold R.; Collins B. C.; Röst H. L.; Mann M. diaPASEF: parallel accumulation–serial fragmentation combined with data-independent acquisition. Nat. Methods 2020, 17, 1229–1236. 10.1038/s41592-020-00998-0. 33257825

[ref48] Demichev V.; Messner C. B.; Vernardis S. I.; Lilley K. S.; Ralser M. DIA-NN: neural networks and interference correction enable deep proteome coverage in high throughput. Nat. Methods 2020, 17, 41–44. 10.1038/s41592-019-0638-x. 31768060 PMC6949130

[ref49] Cox J.; Hein M. Y.; Luber C. A.; Paron I.; Nagaraj N.; Mann M. Accurate proteome-wide label-free quantification by delayed normalization and maximal peptide ratio extraction, termed MaxLFQ. Mol. Cell. Proteomics 2014, 13, 2513–2526. 10.1074/mcp.M113.031591. 24942700 PMC4159666

[ref50] Montaser A. B.; Järvinen J.; Löffler S.; Huttunen J.; Auriola S.; Lehtonen M.; Jalkanen A.; Huttunen K. M. L-Type Amino Acid Transporter 1 Enables the Efficient Brain Delivery of Small-Sized Prodrug across the Blood-Brain Barrier and into Human and Mouse Brain Parenchymal Cells. ACS Chem. Neurosci. 2020, 11, 4301–4315. 10.1021/acschemneuro.0c00564. 33228353

[ref51] Watsky M. A.; Jablonski M. M.; Edelhauser H. F. Comparison of conjunctival and corneal surface areas in rabbit and human. Curr. Eye Res. 1988, 7, 483–486. 10.3109/02713688809031801. 3409715

[ref52] Olsen T. W.; Sanderson S.; Feng X.; Hubbard W. C. Porcine sclera: Thickness and surface area. Invest. Ophthalmol. Vis. Sci. 2002, 43, 2529–2532. 12147580

[ref53] Ljubimov A. V.; Atilano S. R.; Garner M. H.; Maguen E.; Nesburn A. B.; Kenney M. C. Extracellular matrix and Na+,K+-ATPase in human corneas following cataract surgery: Comparison with bullous keratopathy and Fuchs’ dystrophy corneas. Cornea 2002, 21, 74–80. 10.1097/00003226-200201000-00016. 11805512

[ref54] Kumagai A. K.; Glasgow B. J.; Pardridge W. M. GLUT1 glucose transporter expression in the diabetic and nondiabetic human eye. Invest. Ophthalmol. Vis. Sci. 1994, 35, 2887–2894. 8188484

[ref55] del Amo E. M.; Rimpelä A. K.; Heikkinen E.; Kari O. K.; Ramsay E.; Lajunen T.; Schmitt M.; Pelkonen L.; Bhattacharya M.; Richardson D.; Subrizi A.; Turunen T.; Reinisalo M.; Itkonen J.; Toropainen E.; Casteleijn M.; Kidron H.; Antopolsky M.; Vellonen K. S.; Ruponen M.; Urtti A. Pharmacokinetic aspects of retinal drug delivery. Prog. Retin Eye Res. 2017, 57, 134–185. 10.1016/j.preteyeres.2016.12.001. 28028001

[ref56] Bakos É.; Német O.; Kucsma N.; Tőkési N.; Stieger B.; Rushing E.; Tőkés A. M.; Kele P.; Tusnády G. E.; Özvegy-Laczka C. Cloning and characterization of a novel functional organic anion transporting polypeptide 3A1 isoform highly expressed in the human brain and testis. Front Pharmacol 2022, 13, 1–12. 10.3389/fphar.2022.958023. PMC9479004 36120371

[ref57] Huber R. D.; Gao B.; Pfändler M. A. S.; Zhang-Fu W.; Leuthold S.; Hagenbuch B.; Folkers G.; Meier P. J.; Stieger B. Characterization of two splice variants of human organic anion transporting polypeptide 3A1 isolated from human brain. Am. J. Physiol. Cell Physiol. 2007, 292, 795–806. 10.1152/ajpcell.00597.2005. 16971491

[ref58] Pan Q.; Zhang X.; Zhang L.; Cheng Y.; Zhao N.; Li F.; Zhou X.; Chen S.; Li J.; Xu S.; Huang D.; Chen Y.; Li L.; Wang H.; Chen W.; Cai S.; Boyer J.; Chai J. Solute Carrier Organic Anion Transporter Family Member 3A1 Is a Bile Acid Efflux Transporter in Cholestasis. Gastroenterology 2018, 155, 1578–1592.e16. 10.1053/j.gastro.2018.07.031.Solute. 30063921 PMC6221191

[ref59] Bakos É.; Tusnády G. E.; Német O.; Patik I.; Magyar C.; Németh K.; Kele P.; Özvegy-Laczka C. Synergistic transport of a fluorescent coumarin probe marks coumarins as pharmacological modulators of Organic anion-transporting polypeptide, OATP3A1. Biochem. Pharmacol. 2020, 182, 114250 10.1016/j.bcp.2020.114250. 32991865

[ref60] Win A.; Delgado A.; Jadeja R. N.; Martin P. M.; Bartoli M.; Thounaojam M. C. Pharmacological and metabolic significance of bile acids in retinal diseases. Biomolecules 2021, 11, 292 10.3390/biom11020292. 33669313 PMC7920062

[ref61] Daruich A.; Picard E.; Boatright J. H.; Behar-Cohen F. Review: The bile acids urso- and tauroursodeoxycholic acid as neuroprotective therapies in retinal disease. Mol. Vis. 2019, 25, 610–624. 31700226 PMC6817734

[ref62] Mulhern M. L.; Madson C. J.; Kador P. F.; Randazzo J.; Shinohara T. Cellular osmolytes reduce lens epithelial cell death and alleviate cataract formation in galactosemic rats. Mol. Vis. 2007, 13, 1397–1405. 17768385

[ref63] Abdel-Ghaffar A.; Ghanem H. M.; Ahmed E. K.; Hassanin O. A.; Mohamed R. G. Ursodeoxycholic acid suppresses the formation of fructose/streptozotocin-induced diabetic cataract in rats. Fundam Clin Pharmacol 2018, 32, 627–640. 10.1111/fcp.12385. 29863796

[ref64] Verstraelen J.; Reichl S. Expression in Human Corneal Cell Culture Models and Animal Corneal Tissue. Mol. Pharm. 2014, 11, 2160–2171. 10.1021/mp400625z. 24456047

[ref65] Barot M.; Gokulgandhi M. R.; Pal D.; Mitra A. K. Mitochondrial localization of P-glycoprotein and peptide transporters in corneal epithelial cells - Novel strategies for intracellular drug targeting. Exp. Eye Res. 2013, 106, 47–54. 10.1016/j.exer.2012.10.006. 23116562 PMC3545686

[ref66] Kawazu K.; Fujii S.; Yamada K.; Shinomiya K.; Katsuta O.; Horibe Y. Characterization of monocarboxylate uptake and immunohistochemical demonstration of monocarboxylate transporters in cultured rabbit corneal epithelial cells. J. Pharm. Pharmacol. 2013, 65, 328–336. 10.1111/j.2042-7158.2012.01600.x. 23356841

[ref67] Verstraelen J.; Reichl S. Expression analysis of MDR1, BCRP and MRP3 transporter proteins in different in vitro and ex vivo cornea models for drug absorption studies. Int. J. Pharm. 2013, 441, 765–775. 10.1016/j.ijpharm.2012.10.007. 23069916

[ref68] Gunda S. Corneal Absorption and Anterior Chamber. J. Ocul. Pharmacol. Ther. 2006, 22, 465–476. 17238815 10.1089/jop.2006.22.465

[ref69] Majumdar S.; Nashed Y. E.; Patel K.; Jain R.; Itahashi M.; Neumann D. M.; Hill J. M.; Mitra A. K. Dipeptide monoester ganciclovir prodrugs for treating HSV-1-induced corneal epithelial and stromal keratitis: In vitro and in vivo evaluations. Journal of Ocular Pharmacology and Therapeutics 2005, 21, 463–474. 10.1089/jop.2005.21.463. 16386088

[ref70] Anand B. S.; Mitra A. K. Mechanism of corneal permeation of L-Valyl ester of acyclovir: Targeting the oligopeptide transporter on the rabbit cornea. Pharm. Res. 2002, 19, 1194–1202. 10.1023/A:1019806411610. 12240946

[ref71] Anand B. S.; Nashed Y. E.; Mitra A. K. Novel dipeptide prodrugs of acyclovir for ocular herpes infections: Bioreversion, antiviral activity and transport across rabbit cornea. Curr. Eye Res. 2003, 26, 151–163. 10.1076/ceyr.26.3.151.14893. 12815543

[ref72] Xiang C. D.; Batugo M.; Gale D. C.; Zhang T.; Ye J.; Li C.; Zhou S.; Wu E. Y.; Zhang E. Y. Characterization of human corneal epithelial cell model as a surrogate for corneal permeability assessment: metabolism and transport. Drug Metab. Dispos. 2009, 37, 992–998. 10.1124/dmd.108.026286. 19220984

[ref73] Zhang T.; Xiang C. D.; Gale D.; Carreiro S.; Wu E. Y.; Zhang E. Y. Drug transporter and cytochrome P450 mRNA expression in human ocular barriers: Implications for ocular drug disposition. Drug Metab. Dispos. 2008, 36, 1300–1307. 10.1124/dmd.108.021121. 18411399

[ref74] Langford M. P.; Redmond P.; Chanis R.; Misra R. P.; Redens T. B. Glutamate, excitatory amino acid transporters, Xc- antiporter, glutamine synthetase, and γ-glutamyltranspeptidase in human corneal epithelium. Curr. Eye Res. 2010, 35, 202–211. 10.3109/02713680903461489. 20373878

[ref75] Huttunen J.; Peltokangas S.; Gynther M.; Natunen T.; Hiltunen M.; Auriola S.; Ruponen M.; Vellonen K. S.; Huttunen K. M. l-Type Amino Acid Transporter 1 (LAT1/Lat1)-Utilizing Prodrugs Can. Improve the Delivery of Drugs into Neurons. Astrocytes and Microglia, Sci. Rep 2019, 9, 1–12. 10.1038/s41598-019-49009-z. PMC6731241 31492955

[ref76] Rautio J.; Gynther M.; Laine K. LAT1-mediated prodrug uptake: A way to breach the blood-brain barrier?. Ther Deliv 2013, 4, 281–284. 10.4155/tde.12.165. 23442072

[ref77] Miura T.; Tachikawa M.; Ohtsuka H.; Fukase K.; Nakayama S.; Sakata N.; Motoi F.; Naitoh T.; Katayose Y.; Uchida Y.; Ohtsuki S.; Terasaki T.; Unno M. Application of Quantitative Targeted Absolute Proteomics to Profile Protein Expression Changes of Hepatic Transporters and Metabolizing Enzymes During Cholic Acid-Promoted Liver Regeneration. J. Pharm. Sci. 2017, 106, 2499–2508. 10.1016/j.xphs.2017.02.018. 28249806

[ref78] Uchida Y.; Ohtsuki S.; Katsukura Y.; Ikeda C.; Suzuki T.; Kamiie J.; Terasaki T. Quantitative targeted absolute proteomics of human blood-brain barrier transporters and receptors. J. Neurochem 2011, 117, 333–345. 10.1111/j.1471-4159.2011.07208.x. 21291474

[ref79] Jain-Vakkalagadda B.; Dey S.; Pal D.; Mitra A. K. Identification and functional characterization of a Na+-independent large neutral amino acid transporter, LAT1, in human and rabbit cornea. Invest Ophthalmol Vis Sci. 2003, 44, 2919–2927. 10.1167/iovs.02-0907. 12824232

[ref80] Katragadda S.; Talluri R. S.; Pal D.; Mitra A. K. Identification and characterization of a Na+-dependent neutral amino acid transporter, ASCT1, in rabbit corneal epithelial cell culture and rabbit cornea. Curr. Eye Res. 2005, 30, 989–1002. 10.1080/02713680500306439. 16282133

[ref81] Yoneyama D.; Shinozaki Y.; Lu W. L.; Tomi M.; Tachikawa M.; Hosoya K.-I. Involvement of system A in the retina-to-blood transport of l-proline across the inner blood-retinal barrier. Exp. Eye Res. 2010, 90, 507–513. 10.1016/j.exer.2010.01.003. 20074566

[ref82] Umapathy N. S.; Li W.; Mysona B. A.; Smith S. B.; Ganapathy V. Expression and function of glutamine transporters SN1 (SNAT3) and SN2 (SNAT5) in retinal Müller cells. Invest Ophthalmol Vis Sci. 2005, 46, 3980–3987. 10.1167/iovs.05-0488. 16249471

[ref83] Jäger K.; Bönisch U.; Risch M.; Worlitzsch D.; Paulsen F. Detection and regulation of cationic amino acid transporters in healthy and diseased ocular surface. Invest Ophthalmol Vis Sci. 2009, 50, 1112–1121. 10.1167/iovs.08-2368. 18997084

[ref84] Verkman A. S.; Ruiz-Ederra J.; Levin M. H. Functions of aquaporins in the eye. Prog. Retin Eye Res. 2008, 27, 420–433. 10.1016/j.preteyeres.2008.04.001. 18501660 PMC3319433

[ref85] Hamann S.; Zeuthen T.; La Cour M.; Nagelhus E. A.; Ottersen O. P.; Agre P.; Nielsen S. Aquaporins in complex tissues: Distribution of aquaporins 1–5 in human and rat eye. Am. J. Physiol. 1998, 274, C1332–C1345. 10.1152/ajpcell.1998.274.5.c1332. 9612221

[ref86] Patil R.; Wang H.; Sharif N. A.; Mitra A. Aquaporins: Novel targets for age-related ocular disorders. Journal of Ocular Pharmacology and Therapeutics 2018, 34, 177–187. 10.1089/jop.2017.0024. 28632458

[ref87] Vadlapudi A. D.; Vadlapatla R. K.; Mitra A. K. Sodium dependent multivitamin transporter (SMVT): a potential target for drug delivery. Curr. Drug Targets 2012, 13, 994–1003. 10.2174/138945012800675650. 22420308 PMC4406285

[ref88] Janoria K. G.; Hariharan S.; Paturi D.; Pal D.; Mitra A. K. Biotin uptake by rabbit corneal epithelial cells: Role of sodium-dependent multivitamin transporter (SMVT). Curr. Eye Res. 2006, 31, 797–809. 10.1080/02713680600900206. 17038304

[ref89] Janoria K. G.; Boddu S. H. S.; Wang Z.; Paturi D. K.; Samanta S.; Pal D.; Mitra A. K. Vitreal pharmacokinetics of biotinylated ganciclovir: Role of sodium-dependent multivitamin transporter expressed on retina. Journal of Ocular Pharmacology and Therapeutics 2009, 25, 39–49. 10.1089/jop.2008.0040. 19232011 PMC2958449

[ref90] Talluri R. S.; Katragadda S.; Pal D.; Mitra A. K. Mechanism of L-ascorbic acid uptake by rabbit corneal epithelial cells: Evidence for the involvement of sodium-dependent vitamin C transporter 2. Curr. Eye Res. 2006, 31, 481–489. 10.1080/02713680600693629. 16769607

[ref91] Senthilkumari S.; Talwar B.; Dharmaling K.; Ravindran R. D.; Jayanthi R.; Sundaresan P.; Saravanan C.; Young I. S.; Dangour A. D.; Fletcher A. E. Polymorphisms in sodium-dependent vitamin C transporter genes and plasma, aqueous humor and lens nucleus ascorbate concentrations in an ascorbate depleted setting. Exp. Eye Res. 2014, 124, 24–30. 10.1016/j.exer.2014.04.022. 24815519

[ref92] Shui Y. B.; Holekamp N. M.; Kramer B. C.; Crowley J. R.; Wilkins M. A.; Chu F.; Malone P. E.; Mangers S. J.; Hou J. H.; Siegfried C. J.; Beebe D. C. The gel state of the vitreous and ascorbate-dependent oxygen consumption. Archives of Ophthalmology 2009, 127, 475–482. 10.1001/archophthalmol.2008.621. 19365028 PMC2683478

[ref93] Tessier F.; Moreaux V.; Birlouez-Aragon I.; Junes P.; Mondon H. Decrease in vitamin C concentration in human lenses during cataract progression. Int. J. Vitam. Nutr. Res. 1998, 68, 309–315. 9789763

[ref94] Agarwal P.; Rupenthal I. D. In vitro and ex vivo corneal penetration and absorption models. Drug Deliv Transl Res. 2016, 6, 634–647. 10.1007/s13346-015-0275-6. 26762419

[ref95] Naageshwaran V.; Bigonne H.; Gum G.; Malla S.; del Sol C.; Bon C.; Xu X.; Vo A.; Smith W.; O’Reilly Beringhs A.; Kozak D.; Tan M. L.; Babiskin A.; Urtti A.; del Amo E. M.; Ranta V. P. Topical pharmacokinetics of brinzolamide suspensions in rabbits and variability analysis for sample size and design considerations. Int. J. Pharm. 2023, 642, 123183 10.1016/j.ijpharm.2023.123183. 37369289

[ref96] del Amo E. M.; Hammid A.; Tausch M.; Toropainen E.; Sadeghi A.; Valtari A.; Puranen J.; Reinisalo M.; Ruponen M.; Urtti A.; Sauer A.; Honkakoski P. Ocular metabolism and distribution of drugs in the rabbit eye: Quantitative assessment after intracameral and intravitreal administrations. Int. J. Pharm. 2022, 613, 121361 10.1016/j.ijpharm.2021.121361. 34896561

[ref97] Ramsay E.; Ruponen M.; Picardat T.; Tengvall U.; Tuomainen M.; Auriola S.; Toropainen E.; Urtti A.; del Amo E. M. Impact of Chemical Structure on Conjunctival Drug Permeability: Adopting Porcine Conjunctiva and Cassette Dosing for Construction of In Silico Model. J. Pharm. Sci. 2017, 106, 2463–2471. 10.1016/j.xphs.2017.04.061. 28479360

[ref98] Ramsay E.; Hagström M.; Vellonen K.-S.; Boman S.; Toropainen E.; del Amo E. M.; Kidron H.; Urtti A.; Ruponen M. Role of retinal pigment epithelium permeability in drug transfer between posterior eye segment and systemic blood circulation. Eur. J. Pharm. Biopharm. 2019, 143, 18–23. 10.1016/j.ejpb.2019.08.008. 31419586

[ref99] Vellonen K. S.; Häkli M.; Merezhinskaya N.; Tervo T.; Honkakoski P.; Urtti A. Monocarboxylate transport in human corneal epithelium and cell lines. European Journal of Pharmaceutical Sciences 2010, 39, 241–247. 10.1016/j.ejps.2009.12.006. 20035863

[ref100] Nirmal J.; Singh S. B.; Biswas N. R.; Thavaraj V.; Azad R. V.; Velpandian T. Potential pharmacokinetic role of organic cation transporters in modulating the transcorneal penetration of its substrates administered topically. Eye (Basingstoke) 2013, 27, 1196–1203. 10.1038/eye.2013.146. PMC3806579 23846373

[ref101] Vellonen K. S.; Mannermaa E.; Turner H.; Häkli M.; Wolosin J. M.; Tervo T.; Honkakoski P.; Urtti A. Effluxing ABC Transporters in Human Corneal Epithelium. J. Pharm. Sci. 2010, 99, 1087–1098. 10.1002/JPS.21878. 19623615 PMC2906233

[ref102] Dahlin A.; Geier E.; Stocker S. L.; Cropp C. D.; Grigorenko E.; Bloomer M.; Siegenthaler J.; Xu L.; Basile A. S.; Tang-Liu D. D. S.; Giacomini K. M. Gene expression profiling of transporters in the solute carrier and ATP-binding cassette superfamilies in human eye substructures. Mol. Pharmaceutics 2013, 10, 650–663. 10.1021/mp300429e. PMC4032224 23268600

[ref103] Pelis R. M.; Shahidullah M.; Ghosh S.; Coca-Prados M.; Wright S. H.; Delamere N. A. Localization of multidrug resistance-associated protein 2 in the nonpigmented ciliary epithelium of the eye. Journal of Pharmacology and Experimental Therapeutics 2009, 329, 479–485. 10.1124/jpet.108.149625. 19201990 PMC2672870

[ref104] Gao B.; Vavricka S. R.; Meier P. J.; Stieger B. Differential cellular expression of organic anion transporting peptides OATP1A2 and OATP2B1 in the human retina and brain: implications for carrier-mediated transport of neuropeptides and neurosteriods in the CNS. Pflugers Arch 2015, 467, 1481–1493. 10.1007/s00424-014-1596-x. 25132355

[ref105] Kraft M. E.; Glaeser H.; Mandery K.; König J.; Auge D.; Fromm M. F.; Schlötzer-Schrehardt U.; Welge-Lüssen U.; Kruse F. E.; Zolk O. The prostaglandin transporter OATP2A1 is expressed in human ocular tissues and transports the antiglaucoma prostanoid latanoprost. Invest Ophthalmol Vis Sci. 2010, 51, 2504–2511. 10.1167/iovs.09-4290. 20019365

[ref106] Shimada H.; Nakamura Y.; Nakanishi T.; Tamai I. OATP2A1/SLCO2A1-mediated prostaglandin E2 loading into intracellular acidic compartments of macrophages contributes to exocytotic secretion. Biochem. Pharmacol. 2015, 98, 629–638. 10.1016/j.bcp.2015.10.009. 26474801

[ref107] Kubo Y.; Ohtsuki S.; Uchida Y.; Terasaki T. Quantitative Determination of Luminal and Abluminal Membrane Distributions of Transporters in Porcine Brain Capillaries by Plasma Membrane Fractionation and Quantitative Targeted Proteomics. J. Pharm. Sci. 2015, 104, 3060–3068. 10.1002/jps.24398. 25703048

[ref108] Uchida Y.; Goto R.; Takeuchi H.; Łuczak M.; Usui T.; Tachikawa M.; Terasaki T. Abundant expression of OCT2, MATE1, OAT1, OAT3, PEPT2, BCRP, MDR1, and XCT transporters in blood-arachnoid barrier of pig and polarized localizations at CSF- And blood-facing plasma membranes. Drug Metab. Dispos. 2020, 48, 135–145. 10.1124/dmd.119.089516. 31771948

