# Supplementary material for: Transporter Protein Expression of Corneal Epithelium in Rabbit and Porcine: Evaluation of Models for Ocular Drug Transport Study
Source: Mol Pharm. 2024 May 29;21(7):3204–17. doi: 10.1021/acs.molpharmaceut.3c01210 (PMC11474527; doi:10.1021/acs.molpharmaceut.3c01210)
Supplement: Supplementary file 2 — mp3c01210_si_002.pdf [file mp3c01210_si_002.pdf]

**Supporting Information**

# Transporter protein expression of corneal epithelium in rabbit and porcine: Evaluation of models for ocular drug transport study

**AUTHOR NAMES**

Eva Ramsay [1,Ψ,*], Ahmed Montaser [2,Ψ], Kanako Niitsu [2], Arto Urtti [1,2], Seppo Auriola [2], Kristiina M. Huttunen [2], Yasuo Uchida [3], Heidi Kidron [1], Tetsuya Terasaki [1]

**AUTHOR ADDRESS**

[1] Drug Research Programme, Division of Pharmaceutical Biosciences, Faculty of Pharmacy, University of Helsinki, 00014 University of Helsinki, Finland.

[2] School of Pharmacy, University of Eastern Finland, Yliopistonranta 1 C, 70211 Kuopio, Finland.

[3] Department of Molecular Systems Pharmaceutics, Graduate School of Biomedical and Health Sciences, Hiroshima University, 1-2-3 Kasumi, Minami-ku, Hiroshima, 734-0037, Japan.

[Ψ] Equal contribution

* Corresponding author: Faculty of Pharmacy, University of Helsinki, 00014 University of Helsinki, Finland. E-mail address: eva.ramsay@helsinki.fi (E. Ramsay), Telephone number: +358 504429112

Transporter protein expression in human, rabbit, and porcine corneal epithelium tissues or primary cells. In some situations, the RNA expression is reported. Protein expression detected by qualitative (literature data) and targeted/global proteomics (present study) methods.

| | Human | Rabbit | | Porcine | |
|---|---|---|---|---|---|
| | Qualitative Protein Expression | Qualitative Protein Expression | Targeted Proteomics / Global Proteomics | Qualitative Protein Expression | Targeted Proteomics / Global Proteomics |
| 4f2hc/ Slc3a1 | +IHC [1] | | +/+ | | +/+ |
| Abca/Abca 1 | | | not studied/+ | | -/+ |
| Aqp0/Mip | Aqp3, 5: +IHC [2] | Aqp0, 1, 2, 4: -IHC; Aqp1, 3, 5: +IHC [3] | +/- | | +/+ |
| Asct1/ Slc1a4 | | RNA detected [4] | +/- | | +/- |
| Asct2/ Slc1a5 | | | +/+ | | +/+ |
| Bcrp/ Abcg2 | +WB, +IHC [5,6] | -WB, -IHC in [7] | -/- | -WB, -IHC [7] | -/- |
| Bsep/ Abcb11 | | | -/- | | -/- |
| Cat1/ Slc7a1 | +IHC [8] | | not studied/+ | | +/+ |
| Crt1/ Slc6a8 | | | +/- | | +/+ |
| Crt2/ Slc16a12 | | | -/+ | | +/- |
| Eaat1/ Slc1a3 | +IHC [1] | | +/+ | | +/+ |
| Eaat2/ Slc1a2 | +IHC [1] | | -/- | | +/+ |
| Eaat3/ Slc1a1 | +IHC [1] | | not studied/- | | -/- |
| Eaat4/ Slc1a6 | +IHC [1] | | -/- | | -/+ |
| Glut1/ Slc2a1 | +IHC [9] | | +/+ | | +/+ |
| Hmit/ Slc2a13 | | | +/+ | | +/+ |
| Insr/ Insr | +IHC [10,11] | | +/+ | | +/+ |
| Lat1/ Slc7a5 | RNA detected [12] | RNA detected [12] | +/+ | | +/+ |
| Mct1/ Slc16a1 | +WB [13] | +ICC [14] | +/+ | | +/+ |
| Mct2/ Slc16a7 | | +ICC [14] | +/- | | +/- |
| Mct4/ Slc16a3 | +WB [13] | +ICC [14] | +/- | | +/- |
| Mct5/ Slc16a4 | | +ICC [14] | -/- | | -/- |
| Mdr1/ Abcb1 | | +WB, +IHC [7,15] | +/+ | -WB, -IHC [7] | -/+ |
| Mrp1/ Abcc1 | +WB, +IHC [5] | +WB, +IHC [16] | +/+ | -WB, -IHC [16] | -/+ |
| Mrp2/ Abcc2 | +WB [17] | +WB, +IHC [16] | -/- | -WB, -IHC [16] | -/- |
| Mrp3/ Abcc3 | -WB, + WB [5] | +WB, -IHC [7] | +/+ | -WB, +IHC [7] | +/+ |

| | | | | | |
|---|---|---|---|---|---|
| Mrp4/ Abcc4 | -WB, +IHC [5,6] | -WB, -IHC [16] | +/+ | +WB, +IHC [16] | +/+ |
| Mrp5/ Abcc5 | +WB, +IHC [5] | +WB, -IHC [16] | -/- | +WB, +IHC [16] | -/+ |
| Na+/K+ ATPase/ Atp1a1, Atp1a2, Atp1a3 | Enzyme activity measured [18] | | +/+ | | +/+ |
| Ntt4/ Slc6a17 | | | -/- | | -/- |
| Oat1/ Slc22a6 | | | -/- | | -/- |
| Oat2/ Slc22a7 | +IHC [6] | | -/- | | +/- |
| Oat3/ Slc22a8 | | | -/- | | -/- |
| Oat4/ Slc22a11 | | | not studied/- | | -/- |
| Oatp1a2/ Slco1a2 | | | -/- | | -/- |
| Oatp1c1/ Slco1c1 | | | -/- | | -/- |
| Oatp2a1/ Slco2a1 | +IHC [19] | | -/- | | -/+ |
| Oatp2b1/ Slco2b1 | +IHC [19] | | -/- | | -/- |
| Oatp3a1/ Slco3a1 | | | +/- | | +/- |
| Oatp4a1/ Slco4a1 | | | not studied/- | | -/- |
| Oatp4c1/ Slco4c1 | | | -/- | | -/- |
| Oatp6a1/ Slco6a1 | | | not studied/- | | -/- |
| Oct1/ Slc22a1 | mRNA in human cornea [20,21] | | -/- | | -/- |
| Oct2/ Slc22a2 | | | -/- | | -/- |
| Oct3/ Slc22a3 | +IHC [6] | | -/- | | -/- |
| Octn1/ Slc22a4 | mRNA in human cornea [20,21] | +IHC [22] | +/- | | -/- |
| Octn2/ Slc22a5 | mRNA in human cornea [20,21] | +IHC [22] | +/+ | | -/- |
| Ost-α/ Slc51a | | | no signal/- | | -/- |
| Ost-β/ Slc51b | | | not studied/- | | -/- |
| Pept1/ Slc15a1 | mRNA in human cornea [20,21] | +WB, +ICC (mitochondria) [23] | -/- | | -/- |
| Pept2/ Slc15a2 | mRNA in human cornea [20,21] | | -/- | | -/- |
| Smit1/ Slc5a3 | | | -/+ | | +/+ |
| Smvt/ Slc5a6 | | RNA detected [24] | +/+ | | +/+ |
| Snat2/ Slc38a2 | | | +/+ | | +/- |
| Snat3/ Slc38a4 | | | +/- | | +/- |

| | | | | | |
|---|---|---|---|---|---|
| Snat5/ Slc38a5 | | | not studied/- | | -/- |
| Svct1/ Slc23a1 | | | -/- | | -/- |
| Svct2/ Slc23a2 | | RNA detected [25] | not studied/+ | | +/+ |

IHC: immunohistochemical staining, ICC: immunocytochemical staining; WB: western blotting

<u>List of Abbreviations</u>

4f2hc: Amino acid transporter heavy chain
Abca: ATP-binding cassette, sub-family A
Aqp0: Aquaporin 0
Asct: Alanine/serine/cysteine/threonine transporter
Bcrp: Breast cancer resistance protein
Bsep: Bile salt export pump
Cat: Cationic amino acid transporter
Crt: Creatine transporter
Eaat: Excitatory amino acid transporter
Glut: Glucose transporter
Hmit: H(+)-myo-inositol cotransporter
Insr: Insuline receptor
Lat: L-Type amino acid transporter
Mct: Monocarboxylate transporter
Mdr: Multidrug Resistance Protein, also known as P-glycoprotein
Mrp: Multidrug resistance-associated protein
$Na^+/K^+$ - ATPase: Sodium/Potassium-Transporting ATPase
Ntt: Neurotransmitter transporter
Oat: Organic anion transporter
Oatp: Organic anion transporting polypeptide
Oct: Organic cation transporter
Octn: Organic cation transporters novel
Ost-a and b: Organic solute transporter, Alpha and Beta subunit
Pept: Peptide transporter
Smit: Sodium/myo-inositol transporter
Smvt: Sodium-dependent multivitamin transporter
Snat: Sodium-coupled neutral amino acid transporter
Svct: Sodium-dependent vitamin C transporter

References:


[1] M.P. Langford, P. Redmond, R. Chanis, R.P. Misra, T.B. Redens, Glutamate, excitatory amino acid transporters, Xc- antiporter, glutamine synthetase, and γ-glutamyltranspeptidase in human corneal epithelium, Curr Eye Res. 35 (2010) 202–211. https://doi.org/10.3109/02713680903461489.

[2] S. Hamann, T. Zeuthen, M. La Cour, E.A. Nagelhus, O.P. Ottersen, P. Agre, S. Nielsen, Aquaporins in complex tissues: Distribution of aquaporins 1-5 in human and rat eye, Am J Physiol Cell Physiol. 274 (1998). https://doi.org/10.1152/ajpcell.1998.274.5.c1332.

[3] B. Bogner, F. Schroedl, A. Trost, A. Kaser-Eichberger, C. Runge, C. Strohmaier, K.A. Motloch, D. Bruckner, C. Hauser-Kronberger, H.C. Bauer, H.A. Reitsamer, Aquaporin expression and localization in the rabbit eye, Exp Eye Res. 147 (2016) 20–30. https://doi.org/10.1016/j.exer.2016.04.013.

[4] S. Katragadda, R.S. Talluri, D. Pal, A.K. Mitra, Identification and characterization of a Na+-dependent neutral amino acid transporter, ASCT1, in rabbit corneal epithelial cell culture and rabbit cornea, Curr Eye Res. 30 (2005) 989–1002. https://doi.org/10.1080/02713680500306439.

[5] K.S. Vellonen, E. Mannermaa, H. Turner, M. Häkli, J.M. Wolosin, T. Tervo, P. Honkakoski, A. Urtti, Effluxing ABC Transporters in Human Corneal Epithelium, J Pharm Sci. 99 (2010) 1087. https://doi.org/10.1002/JPS.21878.

[6] A. Dahlin, E. Geier, S.L. Stocker, C.D. Cropp, E. Grigorenko, M. Bloomer, J. Siegenthaler, L. Xu, A.S. Basile, D.D.S. Tang-Liu, K.M. Giacomini, Gene expression profiling of transporters in the solute carrier and ATP-binding cassette superfamilies in human eye substructures, Mol Pharm. 10 (2013) 650–663. https://doi.org/10.1021/mp300429e.

[7] J. Verstraelen, S. Reichl, Expression analysis of MDR1, BCRP and MRP3 transporter proteins in different in vitro and ex vivo cornea models for drug absorption studies, Int J Pharm. 441 (2013) 765–775. https://doi.org/10.1016/j.ijpharm.2012.10.007.

[8] K. Jäger, U. Bönisch, M. Risch, D. Worlitzsch, F. Paulsen, Detection and regulation of cationic amino acid transporters in healthy and diseased ocular surface, Invest Ophthalmol Vis Sci. 50 (2009) 1112–1121. https://doi.org/10.1167/iovs.08-2368.

[9] A.K. Kumagai, B.J. Glasgow, W.M. Pardridge, GLUT1 glucose transporter expression in the diabetic and nondiabetic human eye, Invest Ophthalmol Vis Sci. 35 (1994) 2887–2894.

[10] P. Naeser, Insulin Receptors in Human Ocular Tissues: Immunohistochemical demonstration in normal and diabetic eyes, Ups J Med Sci. 102 (1997) 35–40. https://doi.org/10.3109/03009739709178930.

[11] E.M. Rocha, D.A. Cunha, E.M. Carneiro, A.C. Boschero, M.J.A. Saad, L.A. Velloso, Insulin, insulin receptor and insulin-like growth factor-I receptor on the human ocular surface, Adv Exp Med Biol. 506 A (2002) 607–610. https://doi.org/10.1007/978-1-4615-0717-8_85.

[12] B. Jain-Vakkalagadda, S. Dey, D. Pal, A.K. Mitra, Identification and functional characterization of a Na+-independent large neutral amino acid transporter, LAT1, in human and rabbit cornea, Invest Ophthalmol Vis Sci. 44 (2003) 2919–2927. https://doi.org/10.1167/iovs.02-0907.

[13] K.S. Vellonen, M. Häkli, N. Merezhinskaya, T. Tervo, P. Honkakoski, A. Urtti, Monocarboxylate transport in human corneal epithelium and cell lines, European Journal of Pharmaceutical Sciences. 39 (2010) 241–247. https://doi.org/10.1016/j.ejps.2009.12.006.

[14] K. Kawazu, S. Fujii, K. Yamada, K. Shinomiya, O. Katsuta, Y. Horibe, Characterization of monocarboxylate uptake and immunohistochemical demonstration of monocarboxylate transporters in cultured rabbit corneal epithelial cells, Journal of Pharmacy and Pharmacology. 65 (2013) 328–336. https://doi.org/10.1111/j.2042-7158.2012.01600.x.

[15] K. Kawazu, K. Yamada, M. Nakamura, A. Ota, Characterization of cyclosporin A transport in cultured rabbit corneal epithelial cells: P-glycoprotein transport activity and binding to cyclophilin, Invest Ophthalmol Vis Sci. 40 (1999) 1738–1744.

[16] J. Verstraelen, S. Reichl, Expression in Human Corneal Cell Culture Models and Animal Corneal Tissue, Mol Pharm. (2014) 2160–2171.

[17] R.M. Pelis, M. Shahidullah, S. Ghosh, M. Coca-Prados, S.H. Wright, N.A. Delamere, Localization of multidrug resistance-associated protein 2 in the nonpigmented ciliary epithelium of the eye, Journal of Pharmacology and Experimental Therapeutics. 329 (2009) 479–485. https://doi.org/10.1124/jpet.108.149625.

[18] W. Ruf, H. Ebel, ATPase in Human Cornea, Pflugers Arch. 366 (1976) 203–210.

[19] M.E. Kraft, H. Glaeser, K. Mandery, J. König, D. Auge, M.F. Fromm, U. Schlötzer-Schrehardt, U. Welge-Lüssen, F.E. Kruse, O. Zolk, The prostaglandin transporter OATP2A1 is expressed in human ocular tissues and transports the antiglaucoma prostanoid latanoprost, Invest Ophthalmol Vis Sci. 51 (2010) 2504–2511. https://doi.org/10.1167/iovs.09-4290.

[20] C.D. Xiang, M. Batugo, D.C. Gale, T. Zhang, J. Ye, C. Li, S. Zhou, E.Y. Wu, E.Y. Zhang, Characterization of human corneal epithelial cell model as a surrogate for corneal permeability assessment: metabolism and transport, Drug Metabolism and Disposition. 37 (2009) 992–998. https://doi.org/10.1124/dmd.108.026286.

[21] T. Zhang, C.D. Xiang, D. Gale, S. Carreiro, E.Y. Wu, E.Y. Zhang, Drug transporter and cytochrome P450 mRNA expression in human ocular barriers: Implications for ocular drug disposition, Drug Metabolism and Disposition. 36 (2008) 1300–1307. https://doi.org/10.1124/dmd.108.021121.

[22] Q. Garrett, S. Xu, P.A. Simmons, J. Vehige, J.L. Flanagan, M.D. Willcox, Expression and localization of carnitine/organic cation transporter OCTN1 and OCTN2 in ocular epithelium, Invest Ophthalmol Vis Sci. 49 (2008) 4844–4849. https://doi.org/10.1167/iovs.07-1528.

[23] M. Barot, M.R. Gokulgandhi, D. Pal, A.K. Mitra, Mitochondrial localization of P-glycoprotein and peptide transporters in corneal epithelial cells - Novel strategies for intracellular drug targeting, Exp Eye Res. 106 (2013) 47–54. https://doi.org/10.1016/j.exer.2012.10.006.

[24] K.G. Janoria, S. Hariharan, D. Paturi, D. Pal, A.K. Mitra, Biotin uptake by rabbit corneal epithelial cells: Role of sodium-dependent multivitamin transporter (SMVT), Curr Eye Res. 31 (2006) 797–809. https://doi.org/10.1080/02713680600900206.

[25] R.S. Talluri, S. Katragadda, D. Pal, A.K. Mitra, Mechanism of L-ascorbic acid uptake by rabbit corneal epithelial cells: Evidence for the involvement of sodium-dependent vitamin C transporter 2, Curr Eye Res. 31 (2006) 481–489. https://doi.org/10.1080/02713680600693629.
